# Identification of ACHE as the hub gene targeting solasonine associated with non-small cell lung cancer (NSCLC) using integrated bioinformatics analysis

**DOI:** 10.7717/peerj.16195

**Published:** 2023-10-10

**Authors:** Tong Liu, Boke Zhang, Yating Gao, Xingxing Zhang, Jiabing Tong, Zegeng Li

**Affiliations:** 1 Anhui University of Chinese Medicine, Hefei, Anhui, China; 2 Key Laboratory of Xin’An Medicine, Ministry of Education, Hefei, Anhui, China; 3 The First Affiliated Hospital of Anhui University of Chinese Medicine, Hefei, Anhui, China; 4 Key Laboratory of Anhui Provincial Department of Education, Hefei, Anhui, China; 5 Center for Xin’an Medicine and Modernization of Traditional Chinese Medicine, Institute of Health and Medicine, Hefei Comprehensive National Science Center, Hefei, Anhui, China

**Keywords:** Solasonine, ACHE, Bioinformatic, Apoptosis, Anti-inflammatory

## Abstract

**Background:**

Solasonine, as a major biological component of *Solanum nigrum* L., has demonstrated anticancer effects against several malignancies. However, little is understood regarding its biological target and mechanism in non-small cell lung cancer (NSCLC).

**Methods:**

We conducted an analysis on transcriptomic data to identify differentially expressed genes (DEGs), and employed an artificial intelligence (AI) strategy to predict the target protein for solasonine. Subsequently, genetic dependency analysis and molecular docking were performed, with Acetylcholinesterase (ACHE) selected as a pivotal marker for solasonine. We then employed a range of bioinformatic approaches to explore the relationship between ACHE and solasonine. Furthermore, we investigated the impact of solasonine on A549 cells, a human lung cancer cell line. Cell inhibition of A549 cells following solasonine treatment was analyzed using the CCK8 assay. Additionally, we assessed the protein expression of ACHE, as well as markers associated with apoptosis and inflammation, using western blotting. To investigate their functions, we employed a plasmid-based ACHE overexpression system. Finally, we performed dynamics simulations to simulate the interaction mode between solasonine and ACHE.

**Results:**

The results of the genetic dependency analysis revealed that ACHE could be identified as the pivotal target with the highest docking affinity. The cell experiments yielded significant findings, as evidenced by the negative regulatory effect of solasonine treatment on tumor cells, as demonstrated by the CCK8 assay. Western blotting analysis revealed that solasonine treatment resulted in the downregulation of the Bcl-2/Bax ratio and upregulation of cleaved caspase-3 protein expression levels. Moreover, we observed that ACHE overexpression promoted the expression of the Bcl-2/Bax ratio and decreased cleaved caspase-3 expression in the OE-ACHE group. Notably, solasonine treatment rescued the Bcl-2/Bax ratio and cleaved caspase-3 expression in OE-ACHE cells compared to OE-ACHE cells without solasonine treatment, suggesting that solasonine induces apoptosis. Besides, solasonine exhibited its anti-inflammatory effects by inhibiting P38 MAPK. This was supported by the decline in protein levels of IL-1β and TNF-α, as well as the phosphorylated forms of JNK and P38 MAPK. The results from the molecular docking and dynamics simulations further confirmed the potent binding affinity and effective inhibitory action between solasonine and ACHE.

**Conclusions:**

The findings of the current investigation show that solasonine exerts its pro-apoptosis and anti-inflammatory effects by suppressing the expression of ACHE.

## Introduction

Lung cancer is one of the most common malignant cancer and the most predominant cause of death with more than 1.79 million deaths worldwide (Cao et al., 2021; Ferlay et al., 2021; Thandra et al., 2021). According to histologic classification, the two primary types of lung cancer are small-cell lung cancer (SCLC) and non-small cell lung cancer (NSCLC). The majority of lung cancers, or around 80% of cases, are NSCLC, which comprises adenocarcinoma, squamous cell carcinoma, and large cell carcinoma (Nooreldeen & Bach, 2021; Xie et al., 2021). The principal treatment strategies for NSCLC are surgical resection, adjuvant chemotherapy, and adjuvant radiotherapy. However, the 5-year survival rate of lung cancer patients still remains low (Heineman, Daniels & Schreurs, 2017; Liang et al., 2020; Thandra et al., 2021; Zhong et al., 2020). Due to the insufficient efficacy of clinical treatment, it is essential to discover novel drugs and appropriate therapy techniques.

The development of anticancer drugs can make extensive use of plant compounds and their primary active components (Agarwal et al., 2020; Buyel, 2018; Gezici & Şekeroğlu, 2019; Majolo et al., 2019). *Solanum nigrum* L. is a classical natural plant with a broad range of pharmacological effectiveness, including antioxidant and anticancer. The previous study has shown that *Solanum nigrum* L. extracts induced cell apoptosis and autophagy in breast cells (Huang, Syu & Lin, 2010) and hepatocellular carcinoma cells (Nath et al., 2016). In addition, the aqueous extract of *Solanum nigrum* L. has an inhibitory effect on cell migration, and also could suppress aerobic glycolysis function towards human breast cancer MCF7 cells (Ling et al., 2019). Notably, the steroidal glycosides, such as solasonine, which make up the majority of this plant’s biological activity and are members of Solanaceae (Jabamalairaj et al., 2019; Mani et al., 2022).

Solasonine shows the anticancer role that it could against gastric cancer growth and progression (Zhang et al., 2020). Otherwise, solasonine is involved in ferroptosis and activating ubiquitinated degradation of pancreatic cancer cells (Liang et al., 2022). For leukemia cell lines, solasonine shows the inhibition of progression by promoting apoptosis and inducing cell cycle arrest (Zhang et al., 2021). In addition, solasonine is a key component in the production of steroidal anti-inflammatory medications and contraceptives due to its chemical similarities to steroidal hormones (Al Sinani et al., 2016; Al Sinani & Eltayeb, 2017), while also showing significant anticancer properties (Fekry et al., 2019; Pham et al., 2019). Due to its biological properties, solasonine has been reported in the growth suppression of various cancers, including breast carcinoma, liver carcinoma, and stomach adenocarcinoma (Al Sinani & Eltayeb, 2017; Lee et al., 2022; Li et al., 2022; Pham et al., 2019). Nevertheless, the effect and biological mechanism of solasonine on NSCLC have not been extensively studied.

In this study, we extracted the DEGs from the NSCLC dataset in The Cancer Genome Atlas (TCGA) and the solasonine treatment dataset in Gene Expression Omnibus (GEO). Additionally, we employed DeepAffinity, a structure-based AI method, to explore the target protein for solasonine. The intersected genes between the DEGs and the target protein were considered potential markers. Based on the results of genetic dependency analysis and molecular docking, ACHE was selected as the crucial marker for further investigation in relation to solasonine. Subsequently, we employed several bioinformatic techniques, including drug sensitivity analysis and functional enrichment analysis, to unravel the association between ACHE and solasonine. Then, we evaluated the cell inhibition of A549 cells after solasonine treatment by CCK8 assay and the protein expression of ACHE, as well as the apoptosis-related and inflammation-related markers by western blotting. To support the claim that ACHE mediates the anticancer effects of solasonine on apoptosis and inflammation, we conducted experiments using solasonine in A549 cells with an ACHE overexpressing plasmid. Cells transfected with the ACHE overexpression plasmid were collected as the OE-ACHE group, while cells transfected with an empty vector served as the vector group. Finally, we performed dynamics simulations to simulate the interaction mode between solasonine and ACHE. Our findings indicate that solasonine exerts pro-apoptotic and anti-inflammatory effects by downregulating ACHE expression in A549 cells. These results unveil the distinct and critical regulatory mechanism between solasonine and ACHE, offering valuable insights for the exploration of novel therapeutic interventions for NSCLC.

## Materials and Methods

### Data collection and processing

From the PubChem database, we obtained compound solasonine (PubChem CID:537159) with the SDF file of 2D structure and the character set of canonical Simplified Molecular-Input Line-Entry System (SMILES). Moreover, the 3D structure of this compound was identified and optimized *via* the RDKit (Version Q1 2022) tool.

Based on TCGA (Version v32.0) database, NSCLC samples including LUAD (Lung adenocarcinoma) and LUSC (Lung squamous cell carcinoma) datasets were downloaded with the transcriptomic data and corresponding clinical information. The gene expression was transformed into the Reads Per Kilo bases per Million read (rpkm) with the log2 scale values. The normalizeBetweenArrays function from limma R package (Version 3.42.2) was performed to correct expression values. Meanwhile, we applied the sva R package (Version 3.34.0) to integrate and remove batch effects of datasets.

From the GEO database, we collected a public transcriptomic dataset with the solasonine treatment (Accession ID: PRJNA669277). However, this dataset only contains six SRA experiments with the raw sequence reads, while the transcriptomic values are unavailable. Thus, the gene annotation process for the sequence reads would be necessary. First, FastQC (Version 0.11.9) was utilized to do the quality control checks on raw sequence data. Then, the Trim Galore (Version 0.6.5) tool was applied for the adapter trimming and automatic base quality. At last, we performed the Salmon (Version 0.31.1) software to quantify the expression of transcripts for the RNA-seq data.

The Dependency Map (DepMap) is a free online resource that provides cancer dependencies data of various tumor cell lines to discover cancer vulnerabilities (Tsherniak et al., 2017). From the DepMap portal (Version 22Q1), we collected the genetic dependency scores of CRISPR (808 cell lines) and RNAi (789 cell lines) databases, the cellular models of gene mutation and expression, and the sample info of cell lines. Finally, 158 NSCLC cell lines were chosen for further exploration.

The goal of Genomics of Drug Sensitivity in Cancer (GDSC) is to enhance cancer therapies by identifying therapeutic biomarkers *via* the execution of a thorough genomic drug screen (Yang et al., 2013). From the GDSC database (Version 8.4), we obtained the IC50 and AUC values of drug screening, the detained information of all compounds screened (518 drugs), and the genomic data (gene mutation and expression) of cell lines. Notably, 106 cell lines had the consistent NSCLC subtype with the chosen lines from the DepMap database.

### Target prediction for solasonine

Through the use of affinity scores and a structure-based approach, it is feasible to anticipate how a drug and a protein will interact (Grinter & Zou, 2014; Lionta et al., 2014). DeepAffinity, as an artificial intelligence(AI)-based method with unified recurrent and convolutional neural networks, could investigate the compound–protein relationship *via* the sequence data of compound and protein (Karimi et al., 2019). Here, we used the DeepAffinity algorithm to search the potential targets of solasonine and identified the SMILES string of the drug as input data to assess the drug’s affinity scores with numerous proteins.

### Marker genes analysis

For the transcriptomic datasets, we collected a total of 1,145 samples in NSCLC (1,037 tumor and 108 normal patients) and six samples in the solasonine experiment (three positive control and three untreated control). We compared the sample groups in each dataset to identify differentially expressed genes (DEGs) *via* limma R package. Notably, the thresholds were defined as |log2FC| > 2 and adjusted *p*-value < 0.01 in NSCLC, while these parameters would be defined as |log2FC| > 1 and adjusted *p*-value < 0.05 in the solasonine experiment, respectively.

To further explore the gene markers, the intersection of these markers was evaluated among the predicting targets of solasonine, the DEGs between tumor and normal samples of NSCLC, and the DEGs between positive and untreated control of solasonine experiment. Then, we performed the Venn plotting for the marker visualization *via* the VennDiagram R package (Version 1.7.1).

### Genetic dependency and drug-sensitive analysis

According to the knockout data of CRISPR and RNAi in NSCLC cell lines, we applied the correction analysis with the expression levels and knockout effect scores of the aforementioned markers in “Marker Genes Analysis”. Notably, CRISPR scores under 0 mean the knockout effect of the gene would inhibit tumor proliferation, while scores over 0 show the opposite effect in tumor development. The RNAi scores are identified in the range from 0 to 1, while the scores closer to the number 1 indicates the powerful effect after gene silencing.

For the drug-sensitive analysis in NSCLC cell lines, we investigated the statistical difference of all compounds with the IC50 and AUC values in high/low levels of ACHE groups. The findings of significant drugs were clustered by their target pathways and visualized with the box plot. Furthermore, we collected the canonical SMILES of these significant drugs and transformed them into the description of Morgan fingerprints to evaluate the structural similarity between these drugs and solasonine through the RDKit tool.

### Survival analysis

Based on the NSCLC samples from the TCGA dataset, the clinical factors, including age, gender, tumor stage, and pack-years smoked, were collected for the survival analysis. Notably, according to the median expression value of ACHE, these samples were divided into high and low-level groups. And the Kaplan-Meier method was utilized to investigate the differences among various clinical factor groups *via* the survminer R package (Version 0.4.6). The log-rank and cox-p values were performed to assess the statistical significance of the prognostic effect in ACHE groups. Additionally, we performed univariate and multivariate cox regression analyses to validate the independence of the clinical features.

### Functional enrichment analysis

The VIPER (Virtual Inference of Protein-activity by Enriched Regulon analysis) methods could evaluate the protein activity through gene expression (Alvarez et al., 2016). In accordance with the gene regulatory network obtained from the aracne.work R package (Version 1.12.0), we investigated the activity difference of proteins among ACHE groups *via* the viper R package (Version 1.20.0). Through the Database for Annotation Visualization and Integrated Discovery (DAVID) database (Version 2022q1), we performed the biological enrichment analysis (FDR < 0.001) for significant genes. The pheatmap (Version 1. 0.12) and GoPlot (Version 1. 0.2) R packages were performed to visualize these results.

### Experiments

#### Reagents

The solasonine powder (C45H73NO16, molecular weight: 884.06, HPLC ≥ 98%, CAS: 19121-58-5) was purchased from Beijing Solarbio Science & Technology Company (Beijing, China). Solasonine was dissolved in dimethyl-sulfoxide (DMSO) to obtain a 60 μM stock solution, stored at −20 °C, and diluted in working concentrations before use.

#### Cells and cell culture

Non-small cell lung cancer cell line A549 was obtained from Procell (Wuhan, China) and authenticated with short tandem repeat (STR) profiling. The A549 cells were grown in F12K Medium (Gibco, Billings, MT, USA) with 10% fetal bovine serum (Gibco, Billings, MT, USA), 1% penicillin, and streptomycin (Beyotime, Shanghai, China). Cells were incubated at 37 °C in a humidified atmosphere with 5% CO_2_. The cell line was authenticated through short tandem repeat (STR) profiling by Procell Life Science & Technology (Wuhan, China).

#### Cell proliferation assay

The CCK-8 assay was used to determine cell proliferation. A549 cells were seeded in 96-well plates with 5 × 10^3^/100 μl and cultured overnight. Meanwhiles, cells were treated with 100 μl solasonine of different concentrations (0, 5, 10, 15, 20, 30, and 40 μM), and cultured for 24 or 48 h. Then, CCK-8 solution (10 μl) was added to each well, and the cells were incubated for 2 h at 37 °C. The optical density (OD450) absorption was measured *via* an automatic microplate reader (BioTek, Winooski, VT, USA). Each treatment included six duplicate wells, while the experiment was repeated three times. Additionally, cell viability = (OD[drug] − OD[blank])/(OD[control] − OD[blank]) × 100%. The cell inhibition was identified as 1– cell viability.

#### Western blotting

The differential protein expression of Bax, Bcl-2, cleaved caspase-3, ACHE, IL-1β, TNF-α, p-P38MAPK, and p-JNK in the various groups was determined by Western blotting. Cells were lysed in 100 μl RIPA lysis buffer and PMSF mixture (Beyotime, Shanghai, China) at 4 °C for 15 min. The BCA Protein Assay Kit (Beyotime, Shanghai, China) was used to determine protein concentrations. An equal amount of protein samples was separated by 4–12% SDS-PAGE and then transferred to 0.45 μm PVDF membranes (PMembrane, Beijing, China). After being blocked by 5% non-fat milk for 2 h, membranes were incubated with corresponding primary antibodies to ACHE (Servicebio, Wuhan, China), Bax (Abcam, Waltham, MA, USA), Bcl-2 (Bioss, Beijing, China), Cleaved caspase-3 (CST, San Antonio, TX, USA), IL-1β (Abcam, Waltham, MA, USA), TNF-α (Bioss, Beijing, China), p-P38MAPK (Santa Cruz, CA, USA) and p-JNK (CST, San Antonio, TX, USA) at 4 °C overnight. After that, we utilized the HRP-conjugated secondary antibodies to incubate the PVDF membranes at room temperature, and the imaging system (Analytik Jena, Jena, Germany) and enhanced chemiluminescence detection kit (Biosharp, Hefei, China) to detect the immunoblots.

#### Construction, transfection and grouping of over expressed ACHE plasmid

The mRNA sequence of the ACHE (human) gene was retrieved from the NCBI website. Primers and restriction sites were designed for the NM_000665 transcript of the ACHE gene and selected vectors. The mRNA sequence of the target gene, incorporating the restriction site, was synthesized by Genechem Co., Ltd., (Shanghai, China). The synthesized plasmid underwent DNA sequencing, and the sequencing results were compared with the ACHE sequence in the NCBI database to ensure 100% plasmid sequence fidelity.

A549 cells were randomly divided into three groups: control group, vector group, and overexpressed plasmid group (OE-ACHE). When the cell confluence reached 70–80%, the empty vector and the ACHE overexpression plasmid were separately transfected for 6 h, following the instructions provided with the Lipofectamine 2000 transfection reagent (Thermo Fisher, Waltham, MA, USA). The cells were collected 24 h after changing the medium. Cells transfected with the empty vector were collected as the vector group, while cells transfected with the ACHE overexpression plasmid were assigned to the OE-ACHE group.

#### RT-PCR

Total RNA of the A549 cells was extracted by ESscience RNA-Quick Purification Kit (YiShan Biotech, Shanghai, China), and reversely transcribed to cDNA using the SPARKscript II All-in-one RT SuperMix for qPCR Kit (With gDNA Eraser) (SparkJade, Jinan, China). On a Roche LightCycler480 II PCR system (Roche, Basel, Switzerland), the gene levels were determined using 2×SYBR Green qPCR Mix (SparkJade, Jinan, China) with a reaction condition of 94 °C for 3 min, followed by 40 cycles of 94 °C for 20 s, 55 °C for 20 s, and 72 °C for 30 s. Data were analyzed using the 2^−ΔΔCt^ method with β-actin as control. The ACHE primers were synthesized by Sangon (Shanghai, China). The primers were as follow: ACHE (5′-GTAGACGCTACAACCTTCCA-3′ and 5′-TAGAAGCCACCCCCATAGAT-3′), β-actin (5′-CCCTGGAGAAGAGCTACGAG-3′ and 5′-GGAAGGAAGGCTGGAAGAGT-3′).

### Molecular docking

The crystal structure of proteins were determined from Protein Data Bank (PDB) database. The compound solasonine with an optimized 3D structure was identified as the specific antagonist of proteins for ligand-receptor docking. Subsequently, the Autodock Vina (Version 1.1.2) was carried out to perform the molecular docking with the parameters of energy_range at four and exhaustiveness at 50. Then, the global search space for molecular docking was identified to cover all the residue structures of these proteins.

Following a series of protein-ligand docking processes, we calculated the binding affinities and ranked the scores to choose the suit pose of solasonine in the search space. Furthermore, the docking results were evaluated among activity pockets to extract the ligand with the best overall pose. The ligand-receptor interactions of compound solasonine with protein structure were evaluated by the LigPlot (Version 2.2.5) and PyMOL (Version 1.8.x) software.

### Molecular dynamics simulation

The GROMACS software (Version 2019.6) was utilized to evaluate this ligand-receptor interaction through molecular dynamics simulation. The protein was transformed into the CHARMM36 all-atom force field, while the compound solasonine was uploaded to the online tool, the CHARMM General Force Field (CGenFF) program (https://cgenff.paramchem.org/, Version 4.6), to generate the topology file. The ligand-receptor complex was solvated in a dodecahedron box with SPC water model (spc216.gro file). Subsequently, the molecular dynamics simulation workflow with four specific processes were performed, *i.e*., energy minimization, heating, equilibrium, and simulation. Firstly, constrain the heavy atoms of the complex system and perform 50,000 energy minimization steps with the energy step size at 0.01. For energy optimization, the system was slowly heated to 300 K within 100 ps. Next, the complex system was equilibrated for 100 ps. Finally, we performed 10 times of MD simulations for the equilibrated system, while the simulations were subjected to 50 ns with setting the time-step of 2 fs. The trajectory data was saved every 10 ps. Then, the grmsd package was applied to measure the root-mean-square-deviation (RMSD) variation of the protein-ligand complex backbone. The plot was prepared *via* the xmgrace tool (Version 5.1.25).

To evaluate the binding affinity of the protein-ligand complex, the g_mmpbsa tool (Kumari, Kumar & Lynn, 2014), based on the MM-PBSA method, was performed to calculate the binding free energy for the complex with the following formula:

(1) $$\Delta Gbind = \Delta EMM + \Delta Gsol-{\rm T}\Delta S$$

(2) $$\Delta EMM=\Delta Eelectrostatic + \Delta Evdw$$

(3) $$\Delta Gsol = \Delta G_{PB} + \Delta G_{SA}$$

where MM represents molecular mechanics and sol stands for solvation. TΔS suggests the conformational entropy contribution to determine the loss of flexibility upon binding, which could be neglected in calculation (Shen et al., 2012). ∆EMM is defined as the gas-phase energy consisting of electrostatic Δ*Eelectrostatic* and van der Waals interaction Δ*Evdw*. Meanwhiles, Δ*Gsol* contains two elements, including the electrostatic solvation energy Δ*GPB* and the non-polar solvation free energy Δ*GSA*.

### Statistical analysis

The cell experiments were performed three times in parallel, and the data values were expressed as the mean ± standard deviation. The GraphPad Prism software (Version 9.0.0) was utilized for data analysis. Two-tailed Student’s t-test was performed to determine significant p-values for the comparison of the two groups. One-way ANOVA was applied to compare the significance level in three or more groups. The statistically significant were considered as *p < 0.05, **p < 0.01.

## Results

### Target prediction for solasonine

**Figure 1:**
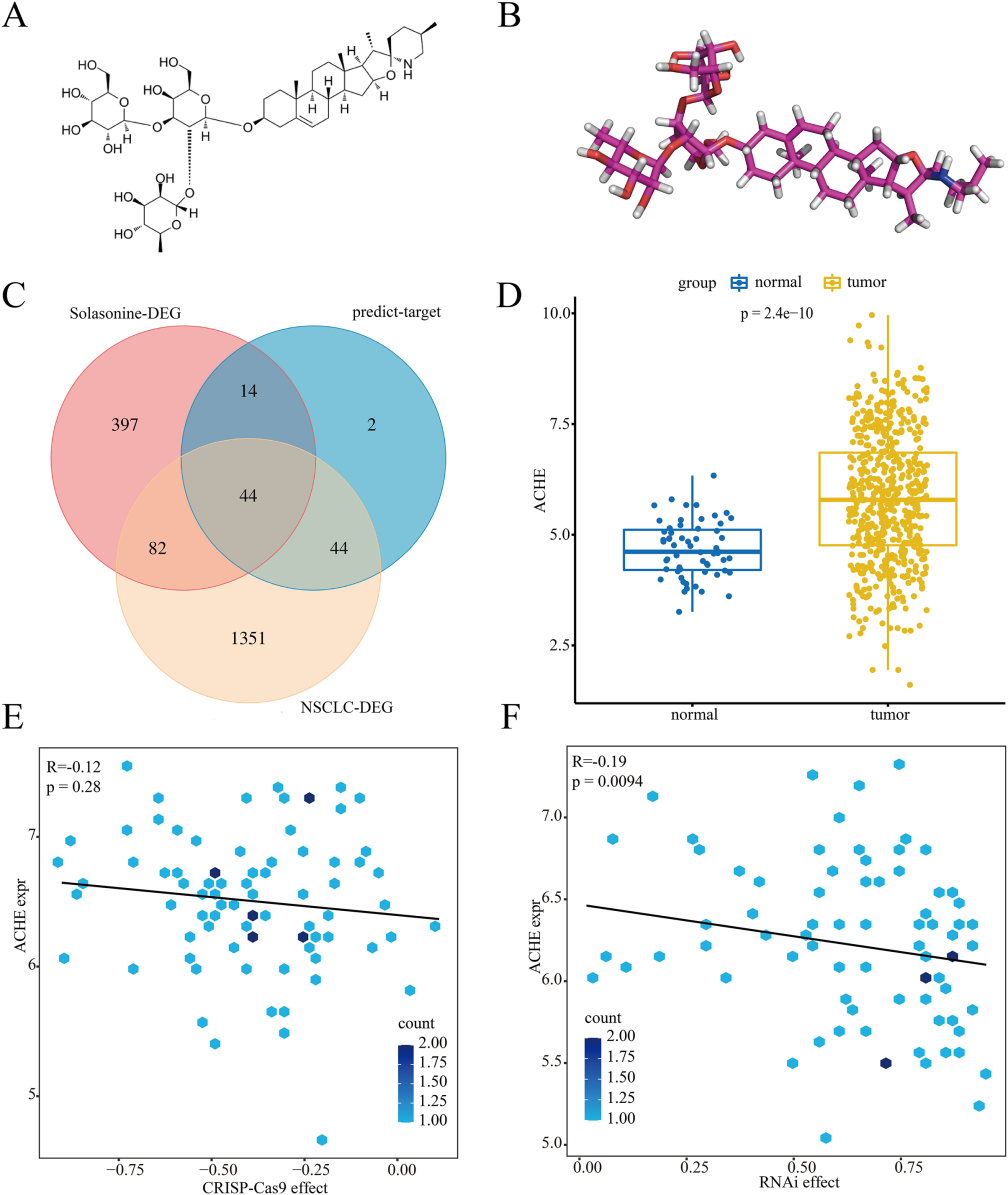
Process of target exploration. The 2D (A) and 3D (B) structures of solasonine. (C) Gene intersection among the DEG in the solasonine treatment dataset, DEG in NSCLC dataset, and predicted targets for solasonine. (D) Expression status of ACHE between tumor and normal groups of NSCLC. (E and F) correlation analysis of ACHE expression in CRISPR (E) and RNAi (F) scores.

As a steroidal glycoalkaloid, solasonine has a special structure with three sugar units, as well as a-l-rhamnopyranose at C-2 or a hydroxyl group on the steroidal backbone (Ding et al., 2013). Figures 1A and 1B suggest the 2D and 3D structures of solasonine. To further investigate the potential relationship between solasonine and NSCLC, we performed a series of bioinformatics exploration processes. As shown in Fig. 1C, we extracted 1,521 significantly expressed genes in the NSCLC dataset and 537 significant genes in the solasonine treatment dataset. Meanwhile, to improve the effectiveness of target prediction for solasonine, we utilized the AI-based method DeepAffinity to evaluate the affinity scores with solasonine and collected 104 proteins. Next, we explored the intersection among these results and extracted a total of 44 candidate genes.

In accordance with the knockout data of CRISPR and RNAi in NSCLC cell lines, we assessed these candidate genes and identified seven pivotal markers, *i.e*., ACHE, CUTA, NGLY1, PTPRF, SYNCRIP, TAF13, and TIMM9. And the CRISPR scores of these markers were almost under 0, which means the pulling down process of them would inhibit the proliferation of NSCLC (Figs. 1E and S2). As shown in Figs. 1D and S1, we compared the expression status of these markers in the tumor and normal sample groups from NSCLC, while the results indicated that ACHE (*p* = 2.4E−10) and the other six genes all had significantly high expression levels in tumor group. Additionally, further analysis highlighted that the expression level of ACHE had no clear correlation with CRISPR (R = −0.12, *p* = 0.28) and RNAi (R = −0.19, *p* = 0.0094) scores (Figs. 1E and 1F), while the score mode also appeared in the other markers (Figs. S2 and S3).

Herein, we evaluated the binding affinities of the above markers with solasonine. The crystal structures of these proteins were collected from the PDB database with the corresponding PDB id, including ACHE (1F8U), CUTA (2ZFH), NGLY1 (2CCQ), PTPRF (1LAR), SYNCRIP (6KOR), TAF13 (6MZD), and TIMM9 (2BSK), respectively. The results indicated that ACHE had the best binding performance at −8.9 kcal/mol, compared with CUTA (−7.9 kcal/mol), NGLY1 (−5.2 kcal/mol), PTPRF (−8.7 kcal/mol), SYNCRIP (−7.6 kcal/mol), TAF13 (−8.6 kcal/mol), and TIMM9 (−8.5 kcal/mol). Notably, it was reported that the steroidal glycoalkaloids could inhibit the activity of acetylcholinesterase (Sucha & Tomsik, 2016), while the acetylcholinesterase-inhibitory activity of solasonine and its antitumor mechanism in NSCLC is still unclear. Thus, we decided to further investigate the potential regulation between solasonine and ACHE.

### Biological analysis for ACHE

**Figure 2:**
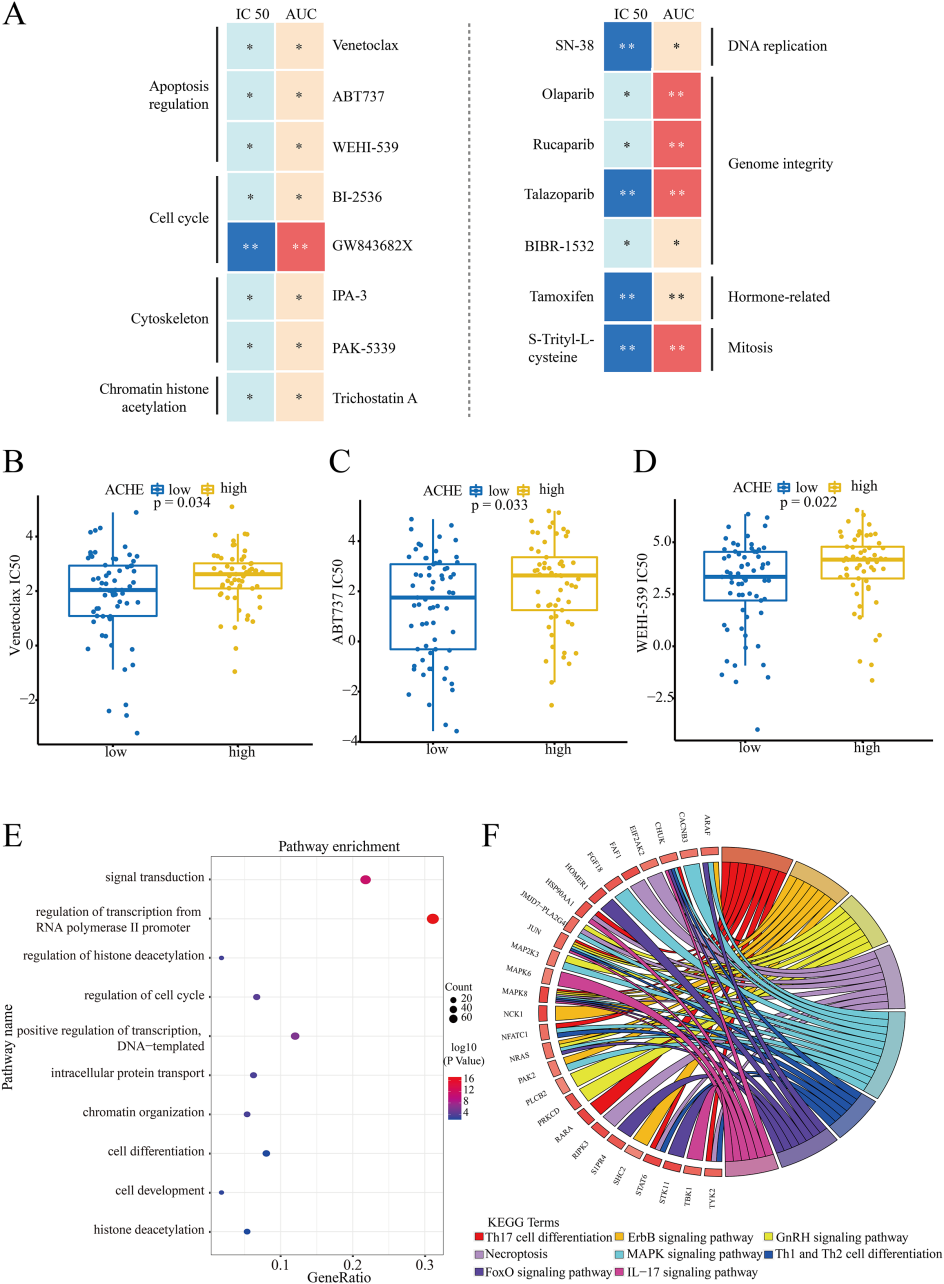
Drug sensitivity of ACHE groups and biological enrichment for marker genes. (A) Compounds with the significant drug-sensitive in IC50 and AUC scores (*p < 0.05, **p < 0.01) and corresponding targeting pathways. (B–D) The differences of IC50 values for these inducers in high/low level groups of ACHE. (B) Result for Venetoclax. (C) Result for ABT737. (D) Result for WEHI-539. (E and F) Biological enrichment analysis for the proteins with significant activity in biological process terms (E) and KEGG pathways (F).

According to the median value of ACHE in NSCLC cell lines, the comparison of IC50 and AUC scores was applied to investigate the drug-sensitive in high/low level groups of ACHE. And the results indicated that a total of 15 compounds had statistical significance in these sensitive scores (*p < 0.05, **p < 0.01) (Fig. 2A). Meanwhile, these compounds could be clustered into eight terms through their targeting pathways, *e.g*., apoptosis regulation, cell cycle, and DNA replication. Then, we evaluated the structural similarity between these significant drugs and solasonine, while it was noticed that Venetoclax, ABT737, and WEHI-539 all appeared in the top-5 similarity scores. The similarity in structures implies similarity in activities or properties (Nikolova & Jaworska, 2003), which means that solasonine may have a potential relationship with ACHE in apoptosis regulation. Notably, as the inducer of apoptosis, Venetoclax (*p* = 0.034, Fig. 2B), ABT737 (*p* = 0.033, Fig. 2C), and WEHI-539 (*p* = 0.022, Fig. 2D) all suggested the significantly high IC50 values in the high expression group of ACHE.

Subsequently, to explore whether high/low expression level of ACHE had an independent prognostic value in the overall survival of NSCLC patients from the TCGA dataset, the univariate and multivariate cox survival analyses were performed to assess different clinical factors. In accordance with the clinical features, *i.e*., age, gender, pack-years smoked, tumor stages, and ACHE groups, the results indicated that the level of ACHE is independent and effective for other clinical characteristics in the overall survival of NSCLC (Table 1).

**Table 1:** Cox regression analysis of ACHE expression groups and clinical characteristics of patients in the NSCLC dataset.

Variables	Univariate cox		Multivariate cox	
	HR (95%) Cl	*p*	HR (95%) Cl	*p*
Age (>66/<=66)	1.243 [0.929–1.663]	0.142	1.211 [0.908–1.627]	0.187
Gender (Male/Female)	1.089 [0.814–1.456]	0.567	0.983 [0.728–1.329]	0.916
Pack years smoked (>38/<=38)	1.015 [0.750–1.372]	0.925	0.995 [0.733–1.350]	0.973
Stage (I/II)	2.365 [1.646–3.398]	1.61E−05	–	–
Stage (I/III)	3.239 [2.221–4.723]	1.01E−08	–	–
Stage (II/III)	1.408 [0.943–2.101]	0.0943	–	–
ACHE	0.614 [0.459–0.821]	9.85E−04	0.617 [0.458–0.830]	1.46E−03

Moreover, we assessed the differences in the relative protein activity between ACHE groups. And the result of the VIPER algorithm demonstrated that a total of 260 significant proteins enriched in these biological process terms, including the regulation of cell cycle, signal transduction, and regulation of histone deacetylation (Fig. 2E). Additionally, these proteins had strong enrichment scores in eight KEGG pathway terms, *e.g*., MAPK, ErbB, and GnRH signaling pathway, respectively (Fig. 2F).

### Solasonine treatment inhibits cell proliferation and regulates the expression of ACHE in A549 cells

**Figure 3:**
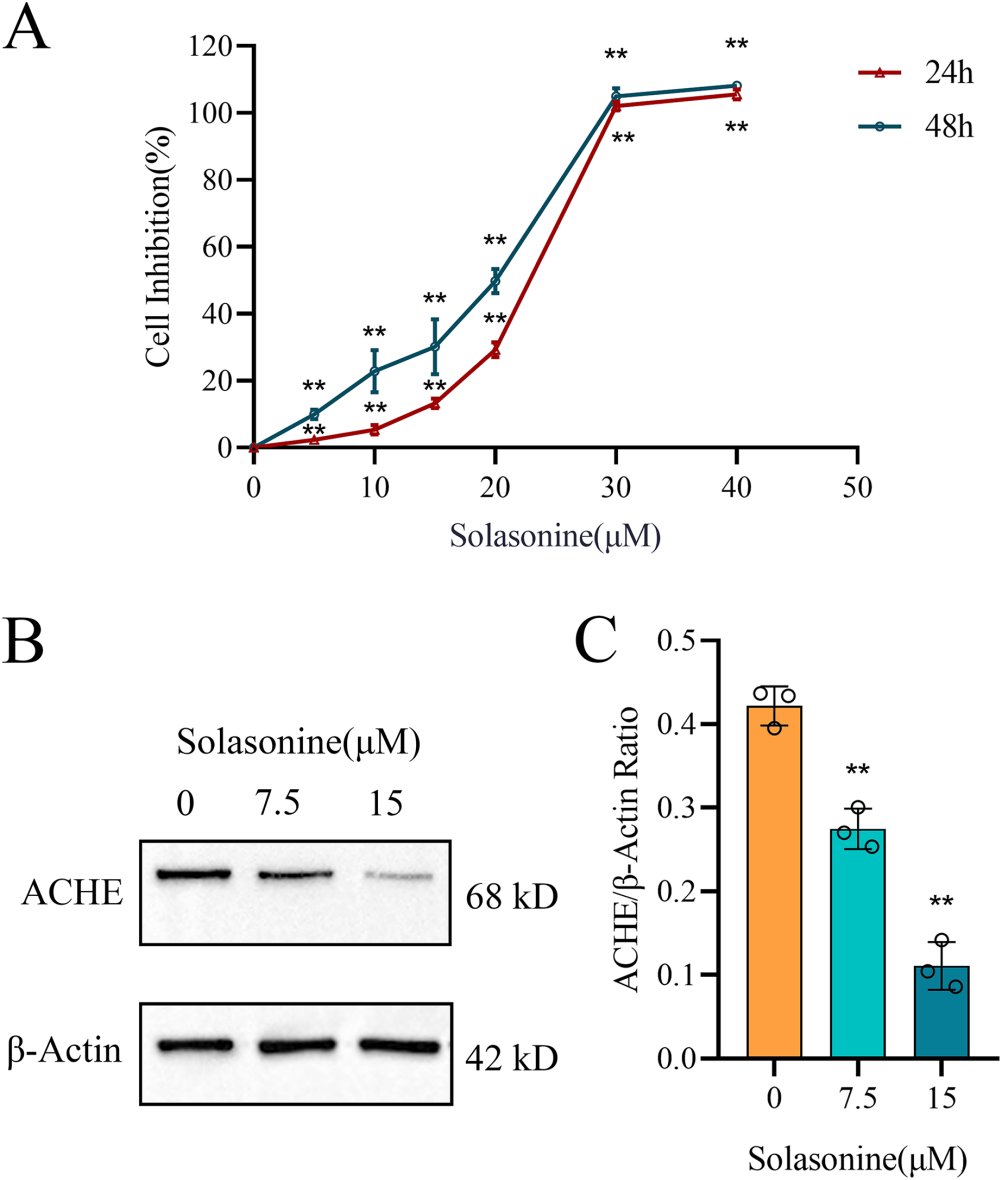
The cell inhibition of A549 cells and the protein levels of ACHE with solasonine treatment. (A) Cell inhibition of A549 treated with different concentrations of solasonine as determined by CCK8 assay. (B and C) Western blotting of ACHE protein expression in A549 and its bar diagram. Data are expressed as mean ± SD (*n* = 3). **p < 0.01 *vs*. the control group (0 µM solasonine).

In this study, A549 cells were subjected to various concentrations of solasonine (0, 5, 10, 15, 20, 30, and 40 μM) for 24 and 48 h to assess cell inhibition using the CCK8 assay. As depicted in Fig. 3A, solasonine concentrations above 20 μM exhibited a highly significant inhibitory effect on cell viability. Specifically, treatment with 15 μM solasonine for 24 h resulted in approximately 15% inhibition of A549 cells growth, whereas treatment with 20 μM solasonine for 24 h led to approximately 30% inhibition. Accordingly, we chose these concentrations (0, 7.5, and 15 μM) for the following experiments. The results demonstrated that solasonine was effective in suppressing the growth of A549 cells.

Furthermore, as illustrated in Figs. 3B and 3C, solasonine treatment markedly reduced the protein expression of ACHE in A549 cells compared to the control group (*p* < 0.01). These results clearly demonstrate the downregulatory effect of solasonine on ACHE expression.

### Solasonine promotes the apoptosis and depresses the inflammation

**Figure 4:**
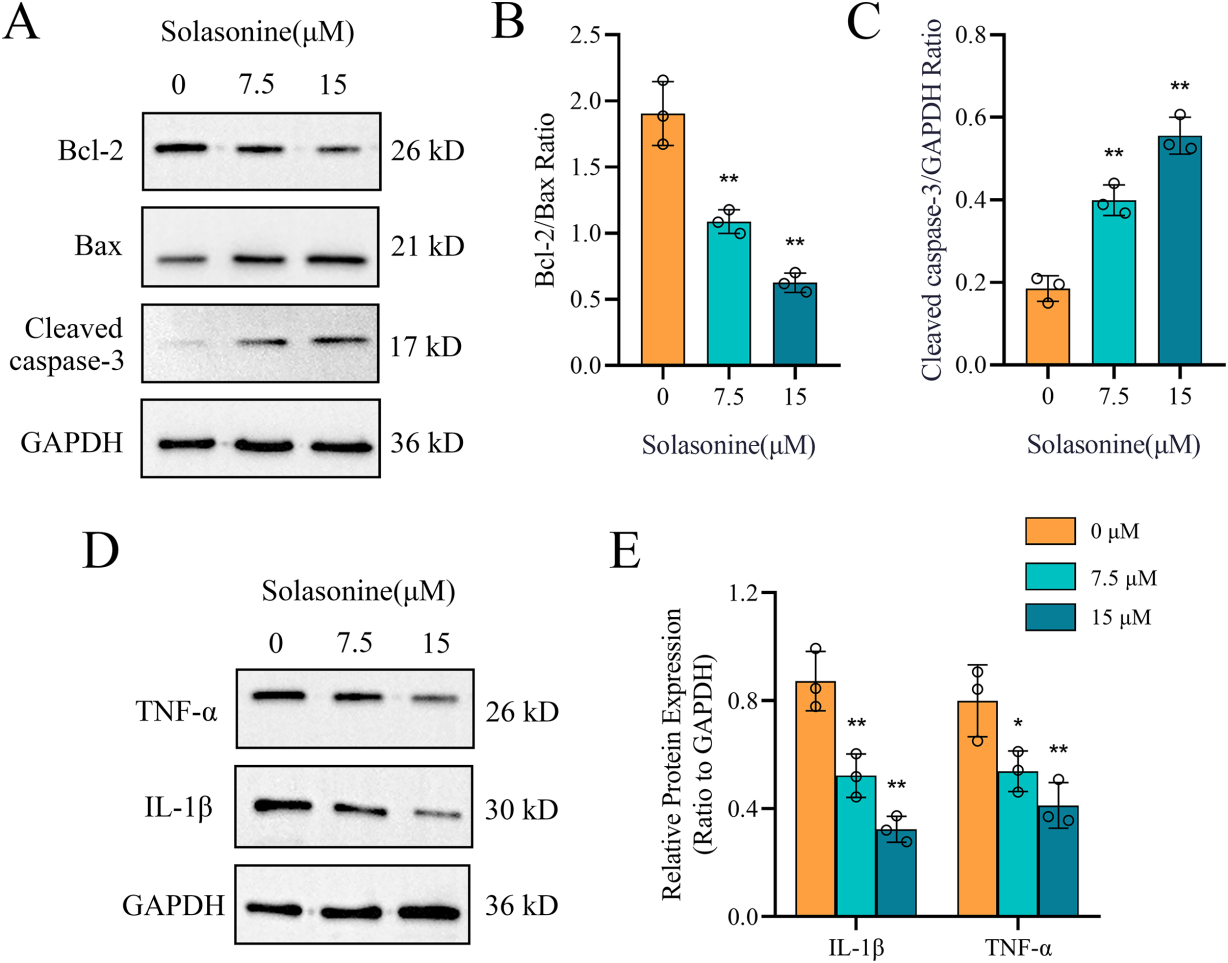
Solasonine influences related protein expressions of apoptosis and inflammation. (A) Protein expression levels of marker proteins of apoptosis, including Cleaved caspase-3, Bax, and Bcl-2 *via* western blotting. (B and C) The bar diagram shows the ratio of Bcl-2/Bax and Cleaved caspase‑3/GAPDH from densitometric analysis of three independent experiments. (D and E) Protein expression levels of IL-1β and TNF-α, and data analysis with bar diagram. Data are expressed as mean ± SD (*n* = 3). *p < 0.05, **p < 0.01 *vs*. the control group (0 µM solasonine).

Clearly, apoptosis is one type of programmed cell death (Obeng, 2020). To further explore the molecular mechanisms through which solasonine regulates the apoptosis, A549 cells were treated with solasonine for 24 h, and examined the expression of proteins associated with apoptosis. Specifically, compared to the control alone, solasonine treatment accelerated the decrease of the Bcl-2/Bax ratio (*p* < 0.01), while the protein expression levels of Cleaved caspase‑3 were increased (*p* < 0.01) (Figs. 4A–4C). Taken collectively, these findings demonstrated that solasonine treatment facilitated apoptosis through the caspase‑dependent pathway in A549 cells.

Inflammation plays a significant role in the formation of tumors, while ACHE is related to inflammation regulation (Vaknine & Soreq, 2020; Zhao et al., 2021). To identify the molecular mechanisms underlying the anti-inflammatory effects of solasonine on A549 cells, the related protein expression levels of IL-1β and TNF-α were measured by western blotting. As shown in Figs. 4D and 4E, the protein expression levels of IL-1β (*p* < 0.01) and TNF-α (*p* < 0.05) revealed the decreased tendency.

### Solasonine affects the processes of apoptosis and inflammation through p38 MAPK pathway

**Figure 5:**
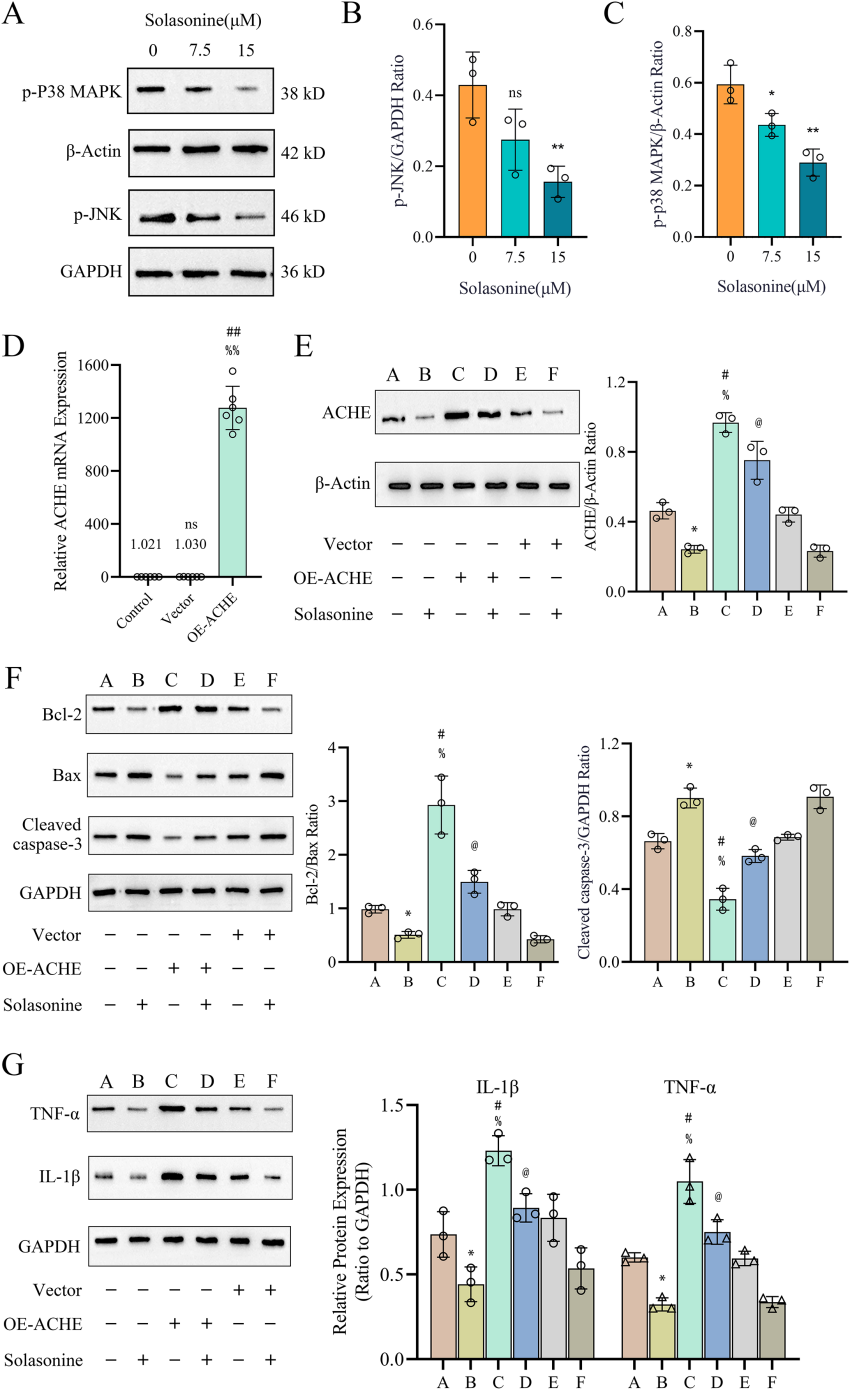
The p38 MAPK pathway contributes to solasonine-induced apoptosis and inflammation in A549 cells. (A–C) Protein expression levels of activated MAPKs, including p-JNK and p-p38 MAPK. Data are expressed as mean ± SD (*n* = 3). *p < 0.05, **p < 0.01 *vs*. the control group (0 μM solasonine). (D) The ACHE mRNA expression in different groups. Data are expressed as mean ± SD (*n* = 6). ^##^*p* < 0.01 *vs*. the control group, ^%%^*p* < 0.01 *vs*. the vector group. (E–G) Protein expression levels of ACHE and marker proteins of apoptosis and inflammation, including Bcl-2, Bax, Cleaved caspase 3, TNF-α, and IL-1β *via* western blotting and data analysis with bar diagram. A549 cells were transfected with ACHE over expression plasmid (lanes C and D) or with empty vector (lanes E and F). Cells were then incubated with (lanes B, D, and F) or without (lanes A, C, and E) 15 µM solasonine for 24 h before harvesting. Data are expressed as mean ± SD (*n* = 3). *p < 0.05 *vs*. the A group, ^#^*p* < 0.05 *vs*. the A group, ^%^*p* < 0.05 *vs*. the E group, ^@^*p* < 0.05 *vs*. the C group.

Subsequently, the activation status of JNK and P38 MAPK was examined. As illustrated in Figs. 5A–5C, treatment of A549 cells with 15 μM solasonine for 24 h significantly suppressed p-JNK (*p* < 0.01) and p-P38 MAPK (*p* < 0.01) compared to the control group. These findings suggest that solasonine inhibits inflammation in A549 cells by modulating the JNK and P38 MAPK signaling pathways.

To further support the statement that ACHE mediates the anticancer effects of solasonine on apoptosis and inflammation, ACHE was overexpressed in the A549 cell line using a plasmid. Western blot analysis was subsequently performed on A549 cells treated with 15 μM solasonine, with or without ACHE overexpression. As shown in Fig. 5D, there was no significant difference in ACHE mRNA expression between the vector group and the control group (*p* < 0.01). However, ACHE mRNA expression was significantly increased in the OE-ACHE group compared to the vector group (*p* < 0.01), indicating successful plasmid transfection. The protein expression of ACHE in OE-ACHE cells was measured after treatment with 15 μM solasonine, revealing a decrease in ACHE protein expression compared to OE-ACHE cells without solasonine treatment (*p* < 0.05) (Fig. 5E).

Furthermore, as observed in Fig. 5F, overexpression of ACHE markedly increased the expression of Bcl-2/Bax and also reduced the expression level of Cleaved caspase 3, an indicator of apoptosis (*p* < 0.05). However, solasonine rescued the Bcl-2/Bax ratio and Cleaved caspase 3 expression in OE-ACHE cells, compared to OE-ACHE cells without solasonine treatment (*p* < 0.05). Regarding the marker proteins of inflammation, as depicted in Fig. 5G, solasonine treatment significantly inhibited the expression of TNF-α and IL-1β compared to the group without solasonine (*p* < 0.05). Interestingly, ACHE overexpression was found to promote the expression of TNF-α and IL-1β in the OE-ACHE group. Importantly, solasonine treatment markedly inhibited the TNF-α and IL-1β expression induced by ACHE overexpression (*p* < 0.05). Therefore, we can conclude that solasonine reduces ACHE expression in A549 cells and regulates the processes of cell apoptosis and inflammation.

### Molecular docking and dynamics simulations analysis

**Figure 6:**
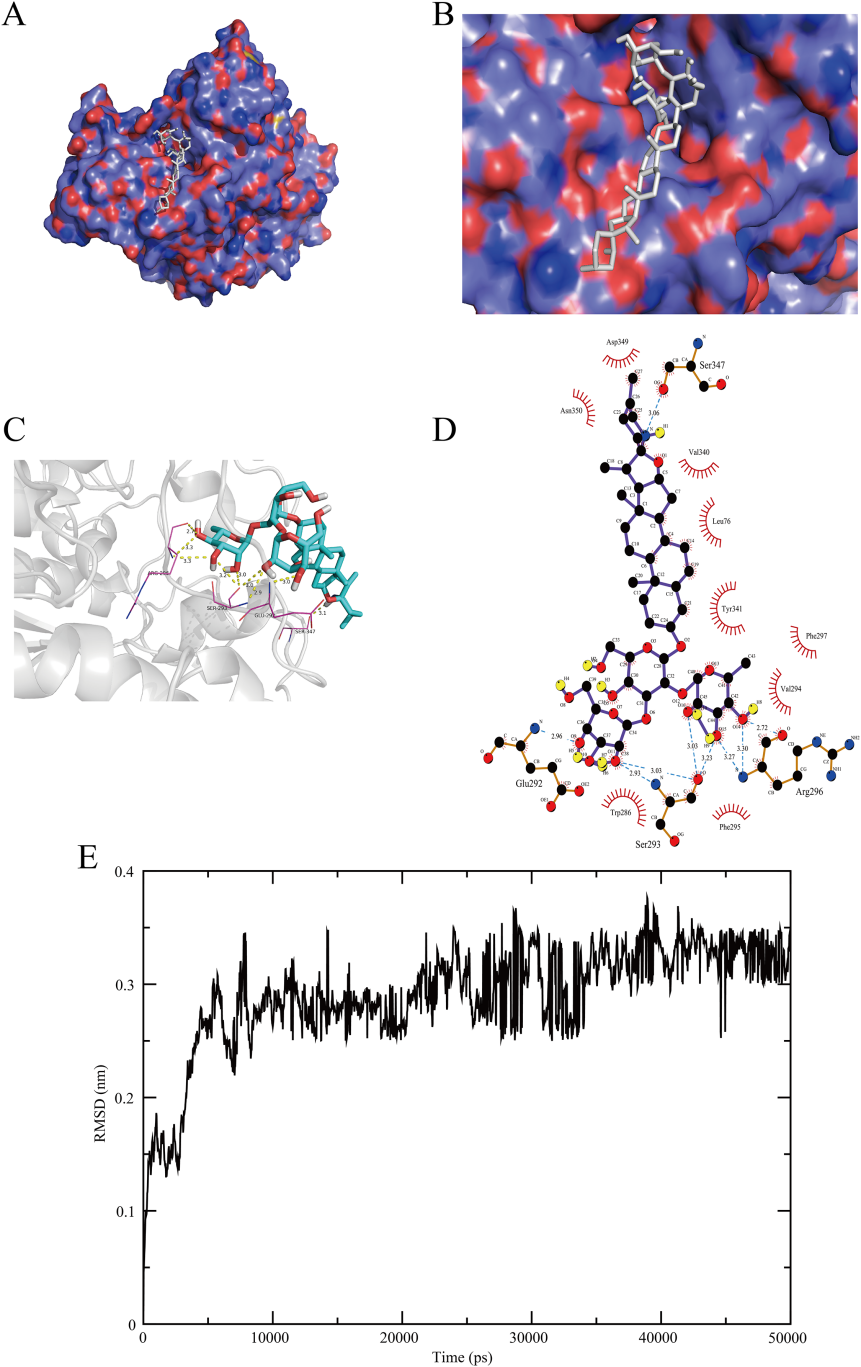
Docking visualization and dynamics simulations of the solasonine-ACHE complex. (A–D) Docking visualization. (E) RMSD profile for the simulated trajectory of the complex system in 50 ns dynamics simulations.

We explored the best activity pocket and displayed the interaction for the solasonine-ACHE complex (Figs. 6A–6D). This complex reflected the great binding effect, showing 9 hydrogen bonds with GLU^292^ (2.96Å), SER^293^ (2.93, 3.03, 3.03, and 3.23Å), ARG^296^ (2.72, 3.27, and 3.30Å), and SER^347^ (3.06Å), respectively. Notably, it was reported that ARG^296^ would be defined as the N-glycosylations site of ACHE protein, while the sequence segment 288 to 303, covering the site of GLU^292^, SER^293^, and ARG^296^, could form the structurally stable disulfide bonds (Cheung et al., 2012).

Additionally, we investigated the spatial conformation of the solasonine-ACHE complex through molecular dynamics simulations. The stability of this complex system was assessed by measuring the root mean square deviation (RMSD) within 50 ns of the simulated trajectory. As shown in Fig. 6E, the RMSD of backbone atoms of the complex system revealed great fluctuation in the initial stage, especially at 0–45 ns. Then, the scores became gentle in subsequent simulations and finally stabilized at 3.3 (±0.28) Å for 10 times of MD simulations. Through the computation with the MM-PBSA method, the detail of the binding free energy about this complex with average values for 10 times simulations is listed in Table 2. The results indicated that the total binding free energy Δ*Gbind* for the solasonine-ACHE complex is −14.445 kcal/mol, suggesting the great binding affinity. In general, according to the aforementioned findings, it is clear that solasonine has the immensely effective structural interaction with ACHE, which provides crucial support for the biological regulation of them.

**Table 2:** Contribution of the various energetic components of the solasonine-ACHE complex to the binding free energy (kcal/mol).

Component	Δ*Evdw*	Δ*Eelectrostatic*	Δ*GPB/GB*	Δ*GSA*	Δ*Egas*(*EMM*)	Δ*Gsol*	Δ*Gbind*
Solasonine-ACHE	−25.379	−2.964	16.005	−2.107	−23.356	13.898	−14.445

**Note:**

*ΔGsol = ΔGPB/GB + ΔGSA; ΔGbind = ΔEvdw + ΔEelectrostatic + ΔGPB/GB + ΔGSA*.

## Discussion

As a steroidal glycoalkaloid, solasonine could be extracted from the traditional natural plant of *Solanum nigrum* L. (Al Sinani & Eltayeb, 2017; Zhu et al., 2020). It is reported that solasonine exhibits the inhibitory action for acetylcholinesterase, as a result of its unique structure (Morillo et al., 2020), but further research and interpretation are required to understand the pharmacological pathways mediated by acetylcholinesterase in NSCLC. In this study, the results of the CCK8 assay show that solasonine is effective in suppressing the growth of A549 cells (Fig. 3A).

To explore the biological targets of solasonine, we screened the DEGs from the NSCLC and solasonine treatment datasets, as well as the predicted targets of solasonine from the AI-based algorithm, while the gene intersection was performed to collect potential targets for solasonine (Fig. 1C). We discovered a total of seven crucial markers which demonstrated possible regulatory connections with solasonine through the genetic dependency analysis with these targets. And ACHE shows the best docking score of solasonine, compared with the other six markers. Additionally, the molecular docking (Figs. 6A–6D) and dynamics simulations (Fig. 6E) suggest the powerful hydrogen interactions between solasonine and the residues of ACHE, which suggests the effectiveness of binding affinity and inhibitory action toward ACHE, and would be chosen for further investigation.

Notably, ACHE has an abnormal expression status in the NSCLC dataset, which suggests the significantly high level in tumor samples compared with normal samples (Fig. 1D). Meanwhile, the knockout data of CRISPR and RNAi in NSCLC cell lines reveals the effective inhibition of ACHE in tumorigenesis (Figs. 1E and 1F). Indeed, it was reported that the lung fibroblast normally does not express or expresses a low level of ACHE, whereas the expression levels tend to be an opposite tendency in different cancer types, such as liver carcinoma and breast carcinoma (Richbart et al., 2021; Xi et al., 2015), demonstrating the heterogeneous biological status of ACHE among cancer types. Consistent with the knockout effect of ACHE, the overexpression of ACHE undergoing apoptosis indicated the inhibition of tumor proliferation (Xu et al., 2014). Similarly, the heterogeneity of ACHE activities among tumors indicates the specific anticancer effect (Lazarevic-Pasti et al., 2017). Thus, the irregular expression and activity status in various tumors reveals the involvement of ACHE in tumorigenesis, while it is necessitated to investigate the important relationship between solasonine and ACHE in NSCLC.

Subsequently, we explored the drug-sensitive data in ACHE groups, and obtained 15 compounds with significant scores (Fig. 2A). It is notable that solasonine shows a higher structural similarity with the apoptosis inducers, which means that solasonine has the potential regulation with apoptosis through targeting ACHE. Indeed, the latest studies have confirmed the apoptosis-inducing effect and the anti-acetylcholinesterase function of solasonine in various human carcinomas (Al Sinani & Eltayeb, 2017; Jin et al., 2020; Zhang et al., 2020). Our result reveals that the solasonine treatment significantly suppresses the protein expression of ACHE in A549 cells (Fig. 3B). Nevertheless, the other study implied that as the marker of apoptosis, the increased expression of ACHE would potentiate chemotherapeutic drug-induced apoptosis (Zhang & Greenberg, 2012). The silencing effect of ACHE shows the prevention of apoptosis activation, which plays a pivotal role in the formation of apoptosis (Pegan et al., 2010; Zhang & Greenberg, 2012). Due to the opposite phenomenon between apoptosis-inducing and the anti-acetylcholinesterase effect of ACHE, we further explored the drug-sensitive data in NSCLC cell lines. The IC50 values of these apoptosis inducers have significant improvement in the high level of ACHE (Figs. 2B–2D), which indicates that the high-expressed ACHE could reduce the effectiveness of the apoptosis drugs in NSCLC. Furthermore, we measured Bcl-2, Bax, and Cleaved caspase-3 through western blotting. And the results suggest that solasonine treatment decreases the ratio level of Bcl-2/Bax and increases the protein expression levels of Cleaved caspase‑3 (Figs. 4A–4C). As seen in Fig. 5F, solasonine rescue the Bcl-2/Bax ratio and Cleaved caspase 3 expression in OE-ACHE cells, compared to OE-ACHE cells without solasonine treatment (*p* < 0.05). These findings indicate that solasonine exerts its pro-apoptosis effects by suppressing the expression of ACHE. Meanwhiles, it is reported that cisplatin with a suitable range of concentrations would inhibit ACHE activity and regulate cell apoptosis (Hassan Baig et al., 2014; Xi et al., 2015). As illustrated above, these findings reveal the heterogeneity of ACHE in tumor regulation and apoptosis, especially in NSCLC.

Notably, the increasing level of ACHE would also regulate the production of inflammatory cytokines and enhance inflammation by inactivating acetylcholine, which roles in the modulation of inflammation (Liu et al., 2015; de Oliveira et al., 2012; Xia et al., 2022). And the ACHE inhibitors suggest pharmacological properties with anti-inflammatory effects (Arikawa et al., 2016; Vaknine & Soreq, 2020). Hence, we examined the expression of proteins associated with inflammation. The finding shows decreasing levels of IL-1β and TNF-α, whereas the A549 cells are treated with solasonine (Fig. 4D). More importantly, it was observed that treated with solasonine markedly inhibited TNF-α and IL-1β expression induced by over expressing ACHE plasmid (*p* < 0.05) (Fig. 5G). It is shown that solasonine could exert the anti-inflammatory effect by suppressing the expression of ACHE. Meanwhile, we found the genes with differential activity in ACHE groups show significant functional enrichment in the MAPK pathway (Fig. 2F). MAPK signaling pathways are an attractive therapeutic target which include JNK and p38 MAPK signaling pathways. Certain inhibitors of MAPK have been used in order to treat cancer by correcting aberrant MAPK signaling (Vergote et al., 2020). We further examined the phosphorylated form of JNK and P38 MAPK, which are involved in inflammation regulation (Li, Hong & Zheng, 2019; Wagner & Nebreda, 2009). The results indicate that solasonine significantly decreases the phosphorylated form of JNK and P38 MAPK (Figs. 5A–5C). Indeed, cancer and inflammation are tightly associated, while some chronic inflammatory diseases would be correlated with a higher risk of cancer development (Munn, 2017). Additionally, p38 could regulate the production of tumor necrosis factor (TNF), interleukin 1 (IL-1), and other cytokines such like COX2 and IL17. Existing studies coincidently report the pro-inflammatory and pro-oncogenic functions in various types of cancer with p38 (Cai et al., 2017; Martínez-Limón et al., 2020). Thus, it is clear that solasonine could exert the anti-inflammatory effect through the inhibition of the production for P38 MAPK.

**Figure 7:**
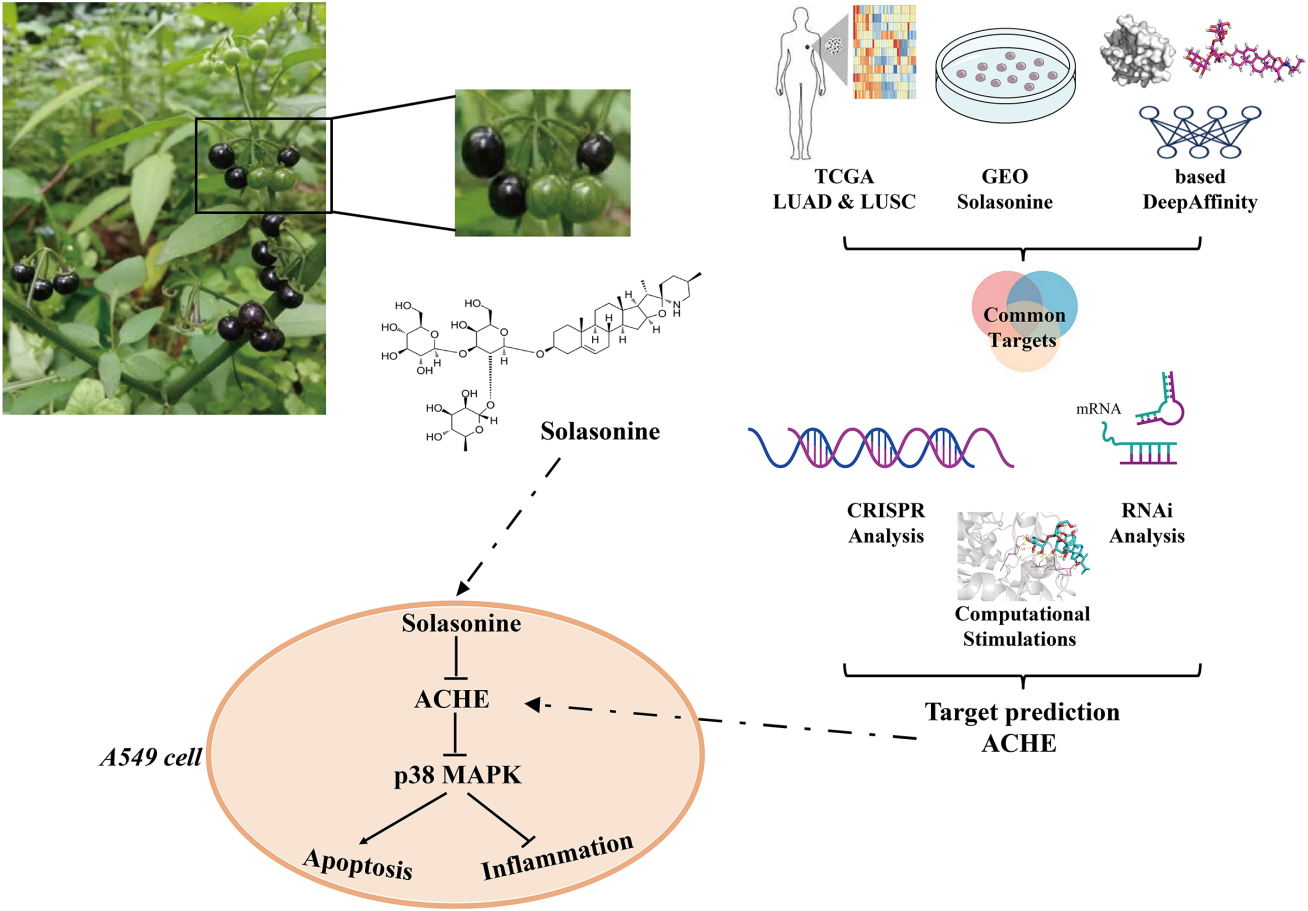
Major processes of this study.

Besides, p38 MAPK cascades are involved in apoptotic mechanisms (Yue & López, 2020). There are many examples of apoptotic processes mediated by p38 MAPK transcriptional regulation. It is reported that p38 MAPK signaling pathway activation could enhance cell survival *via* the suppression of apoptosis (Shao et al., 2016). Using the inhibitor of MAPK/ERK kinase shows an enhancement of peripheral benzodiazepine receptor ligand-induced apoptosis of esophageal cancer cells (Sutter et al., 2004). Therefore, solasonine can be used to affect the apoptosis process through inhibiting p38 MAPK pathway. In general, the aforementioned findings demonstrated that it is reasonable to declare that solasonine, as an ACHE inhibitor, would be effective for tumor treatment through the regulation of cell apoptosis and inflammation by p38 MAPK pathway (Fig. 7).

## Conclusions

In summary, we identified the marker genes from the transcriptome data and AI-based target prediction, and assessed these genes with genetic dependency analysis to obtain significant markers, especially ACHE. Subsequently, we performed a series of bioinformatics explorations for the biological values of ACHE and evaluated these results through cell experiments. Our study indicates that solasonine exerts the pro-apoptosis and anti-inflammatory effect through down-regulating ACHE expression in A549 cells, revealing the dissimilar and critical regulation mode between solasonine and ACHE, which could aid in the investigation of novel medications for the treatment of NSCLC.

## Supplemental Information

10.7717/peerj.16195/supp-1 Supplemental Information 1 The expression status of markers between the tumor and normal sample groups from NSCLC. (A)CUTA. (B)NGLY1. (C)PTPRF. (D)SYNCRIP. (E)TAF13. (F)TIMM9.

10.7717/peerj.16195/supp-2 Supplemental Information 2 The CRISPR scores of markers with expression status. (A)CUTA. (B)NGLY1. (C)PTPRF. (D)SYNCRIP. (E)TAF13. (F)TIMM9.

10.7717/peerj.16195/supp-3 Supplemental Information 3 The RNAi scores of markers with expression status. (A)CUTA. (B)NGLY1. (C)PTPRF. (D)SYNCRIP. (E)TAF13. (F)TIMM9.

10.7717/peerj.16195/supp-4 Supplemental Information 4 Original data.

10.7717/peerj.16195/supp-5 Supplemental Information 5 Analysis code.

## Additional Information and Declarations

### Data Availability

The following information was supplied regarding data availability: The raw data, including coding R, DepMap, TCGA, GEO, GDSC, and files for MD are available at figshare: Liu, Tong (2022). Paper Data. figshare. Dataset. https://doi.org/10.6084/m9.figshare.21789110.v3.
