# Supplementary figures and images for: Identification of ACHE as the hub gene targeting solasonine associated with non-small cell lung cancer (NSCLC) using integrated bioinformatics analysis
Source: PeerJ. 2023 Oct 10;11:e16195. doi: 10.7717/peerj.16195 (PMC10573390; doi:10.7717/peerj.16195)

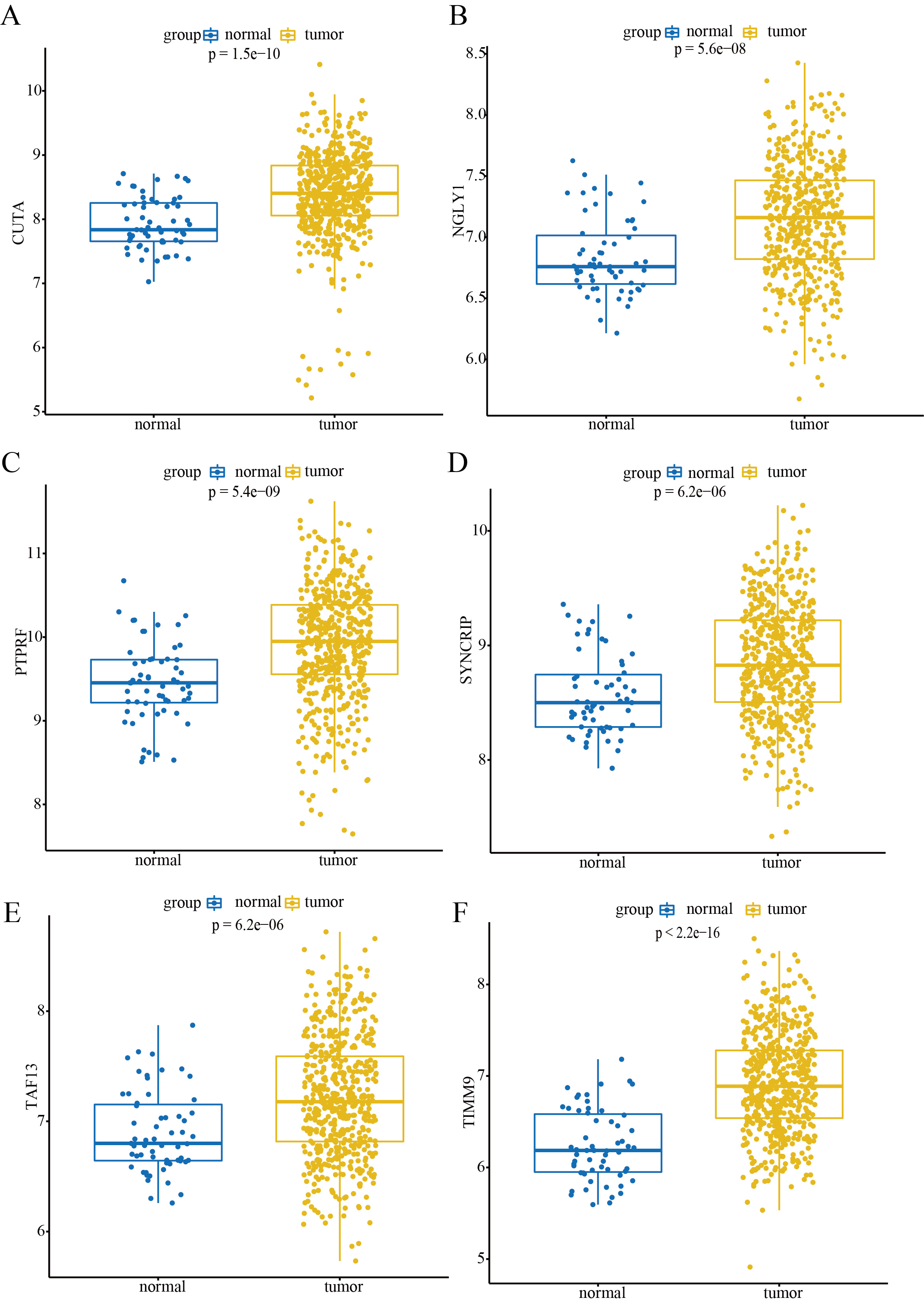

Supplement: Supplemental Information 1 — (A)CUTA. (B)NGLY1. (C)PTPRF. (D)SYNCRIP. (E)TAF13. (F)TIMM9. [file peerj-11-16195-s001.jpg]

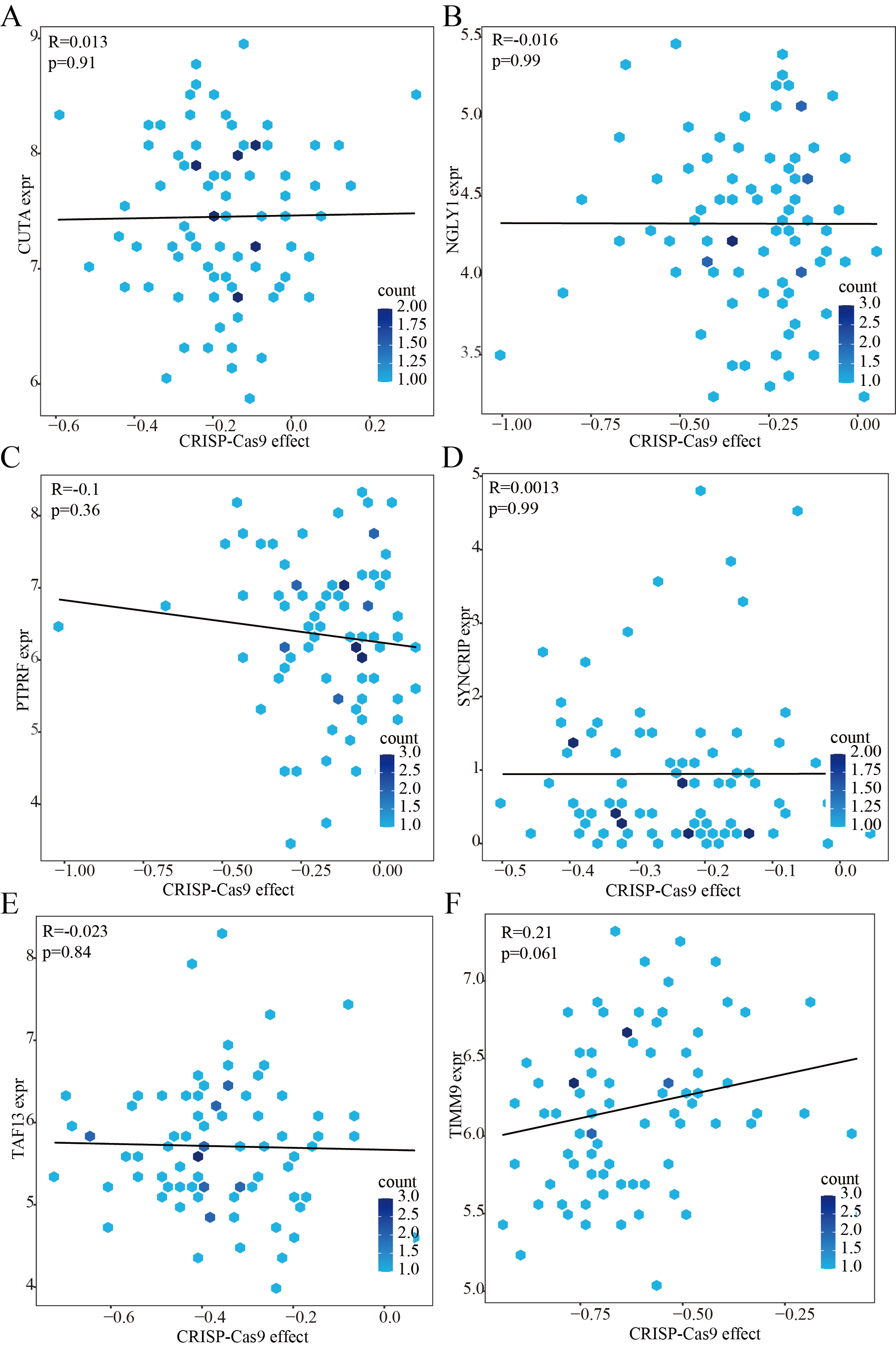

Supplement: Supplemental Information 2 — (A)CUTA. (B)NGLY1. (C)PTPRF. (D)SYNCRIP. (E)TAF13. (F)TIMM9. [file peerj-11-16195-s002.jpg]

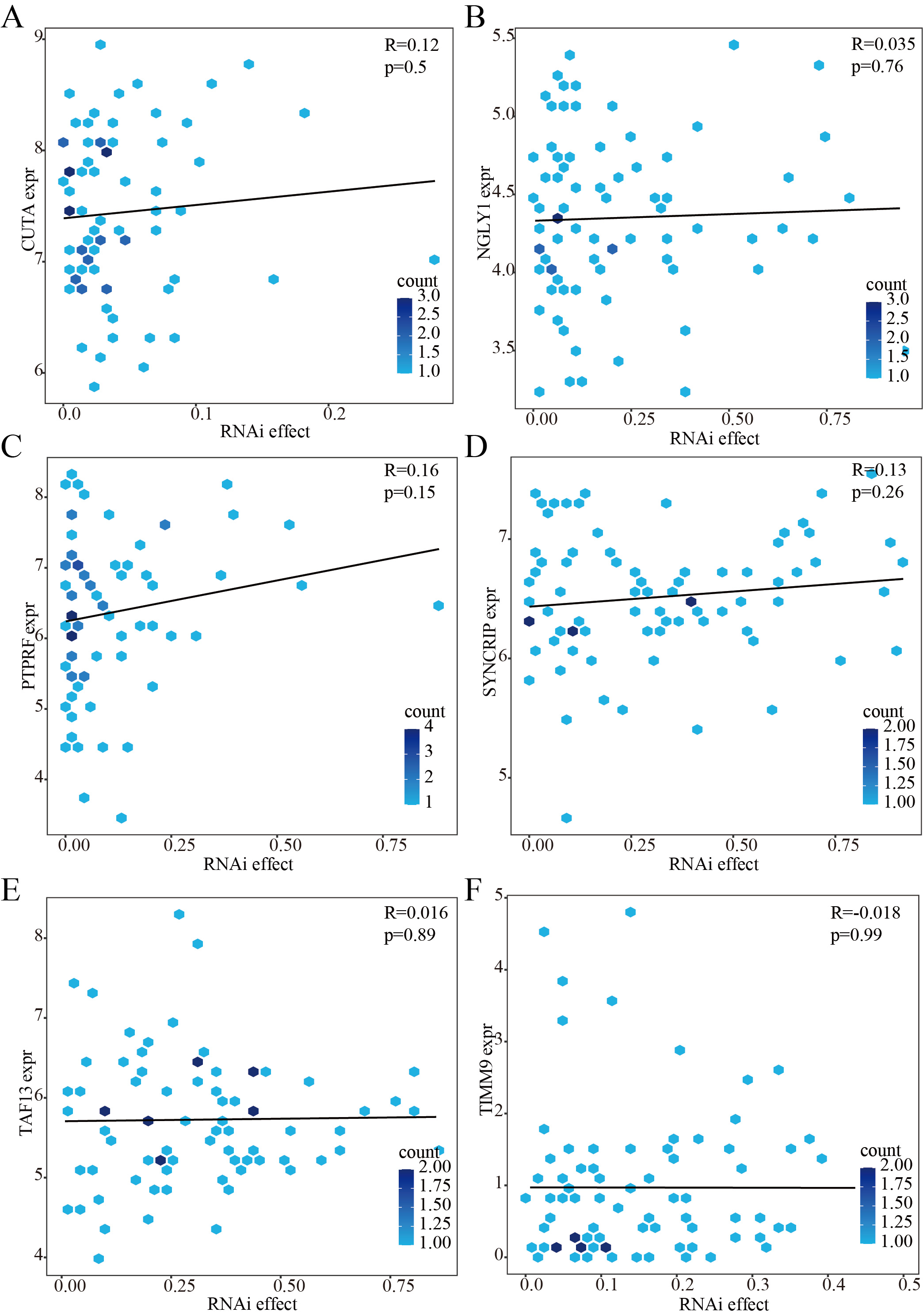

Supplement: Supplemental Information 3 — (A)CUTA. (B)NGLY1. (C)PTPRF. (D)SYNCRIP. (E)TAF13. (F)TIMM9. [file peerj-11-16195-s003.jpg]
