# Supplementary material for: Identification of ACHE as the hub gene targeting solasonine associated with non-small cell lung cancer (NSCLC) using integrated bioinformatics analysis
Source: PeerJ. 2023 Oct 10;11:e16195. doi: 10.7717/peerj.16195 (PMC10573390; doi:10.7717/peerj.16195)
Supplement: Supplemental Information 4 [file peerj-11-16195-s004.pdf]

Figure 3B

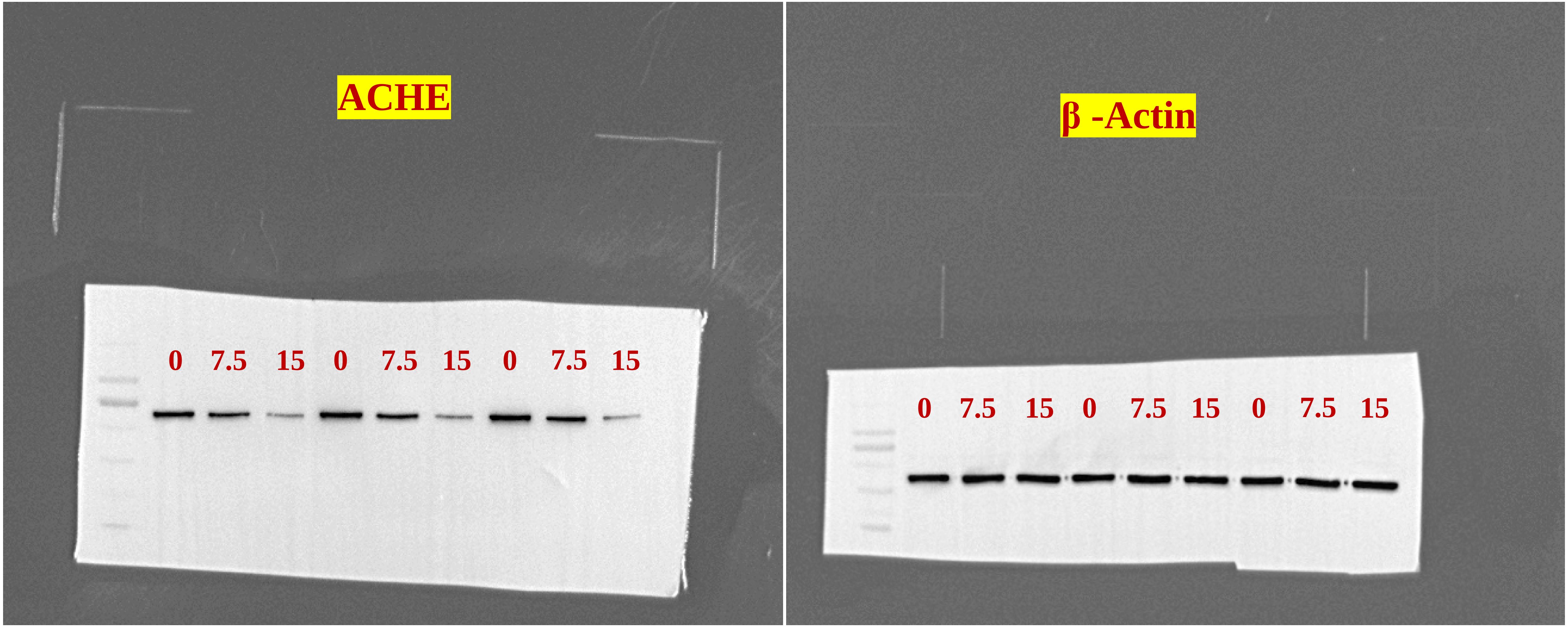

# Figure 4A

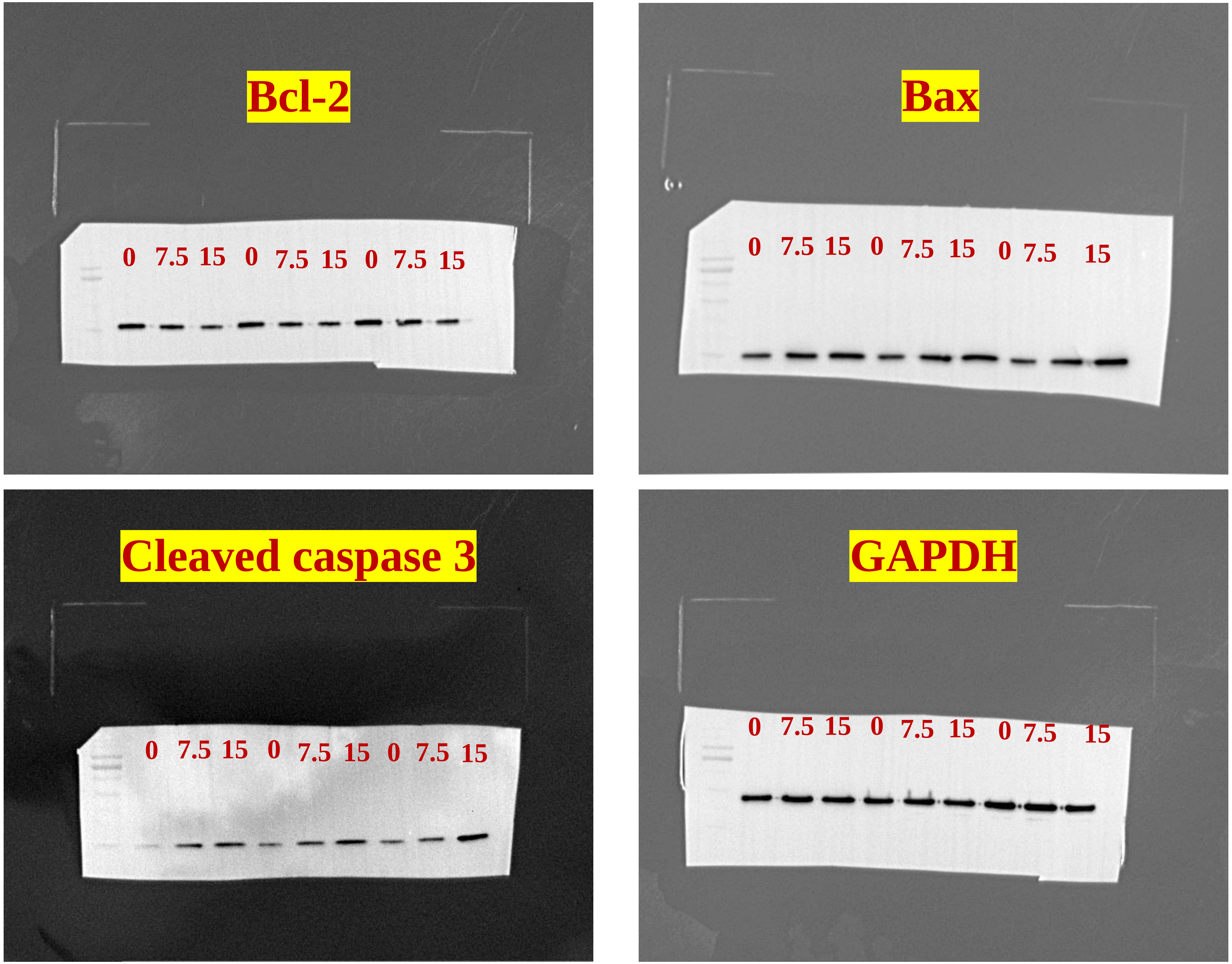
Bcl-2
0 7.5 15 0 7.5 15 0 7.5 15
Bax
0 7.5 15 0 7.5 15 0 7.5 15
Cleaved caspase 3
0 7.5 15 0 7.5 15 0 7.5 15
GAPDH
0 7.5 15 0 7.5 15 0 7.5 15

Figure 4D

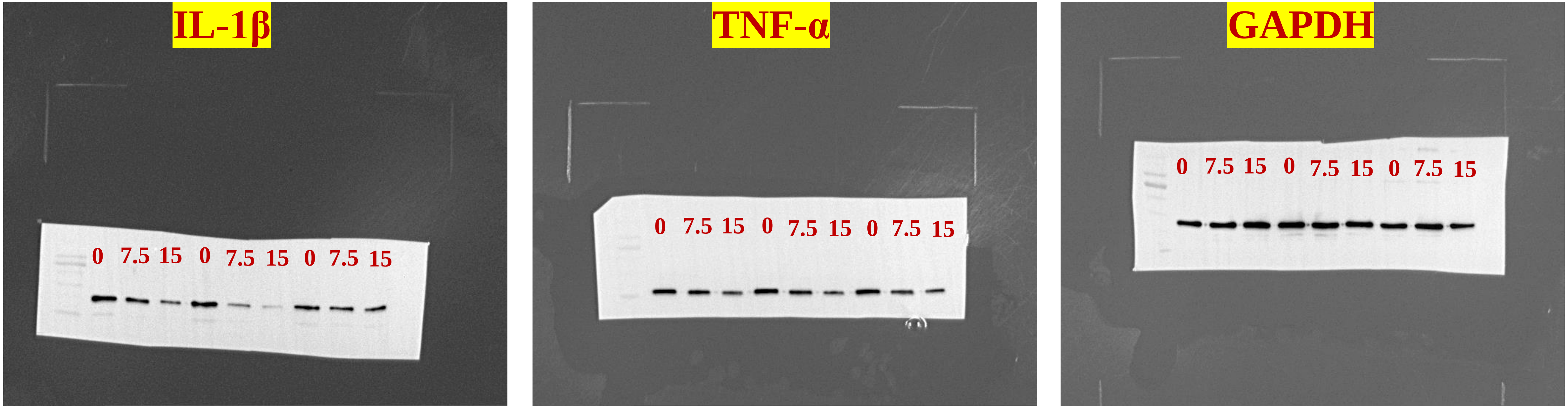
IL-1β
0 7.5 15 0 7.5 15 0 7.5 15
TNF-α
0 7.5 15 0 7.5 15 0 7.5 15
GAPDH
0 7.5 15 0 7.5 15 0 7.5 15

Figure 5A

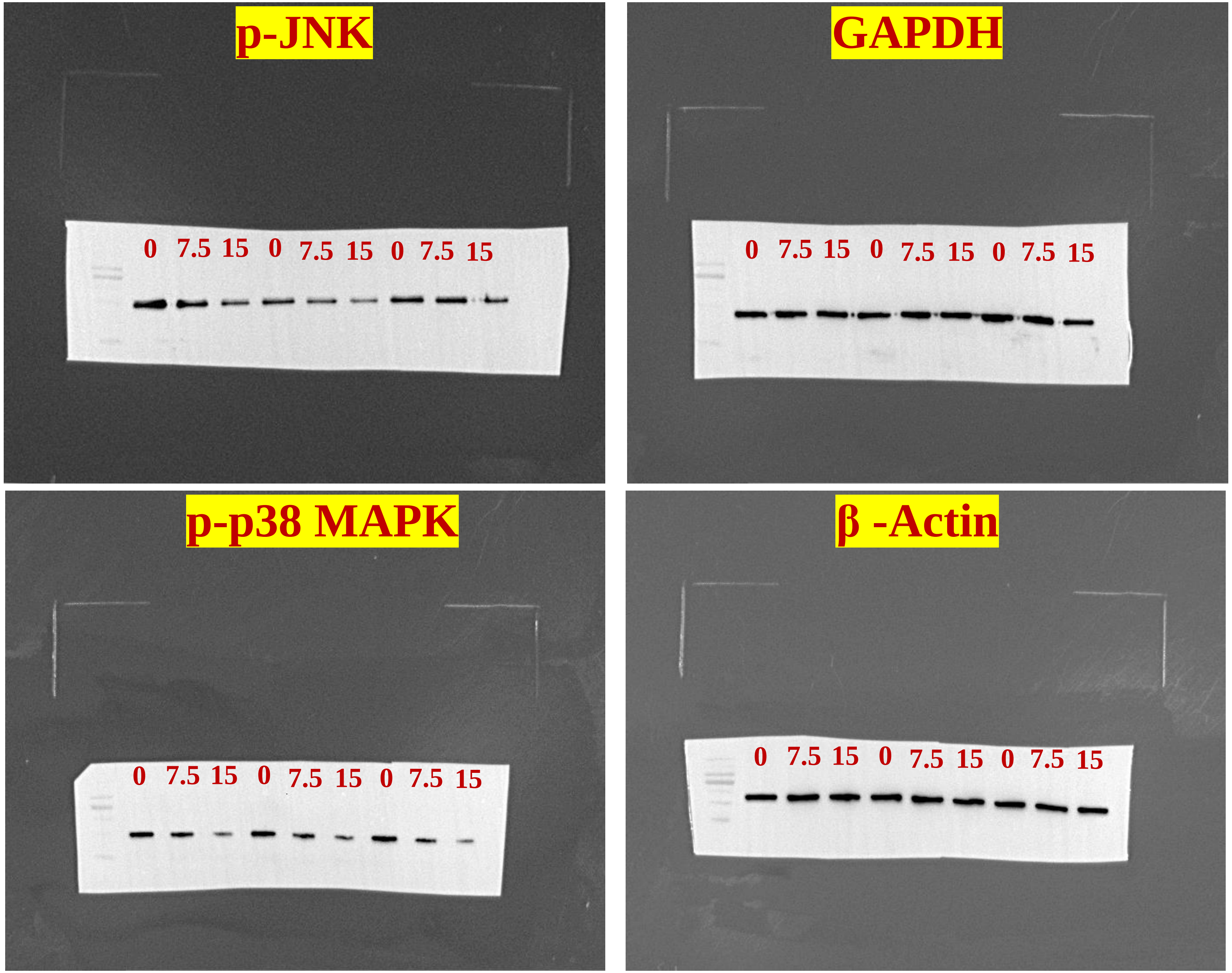
p-JNK
0 7.5 15 0 7.5 15 0 7.5 15
GAPDH
0 7.5 15 0 7.5 15 0 7.5 15
p-p38 MAPK
0 7.5 15 0 7.5 15 0 7.5 15
β -Actin
0 7.5 15 0 7.5 15 0 7.5 15

Figure 5E

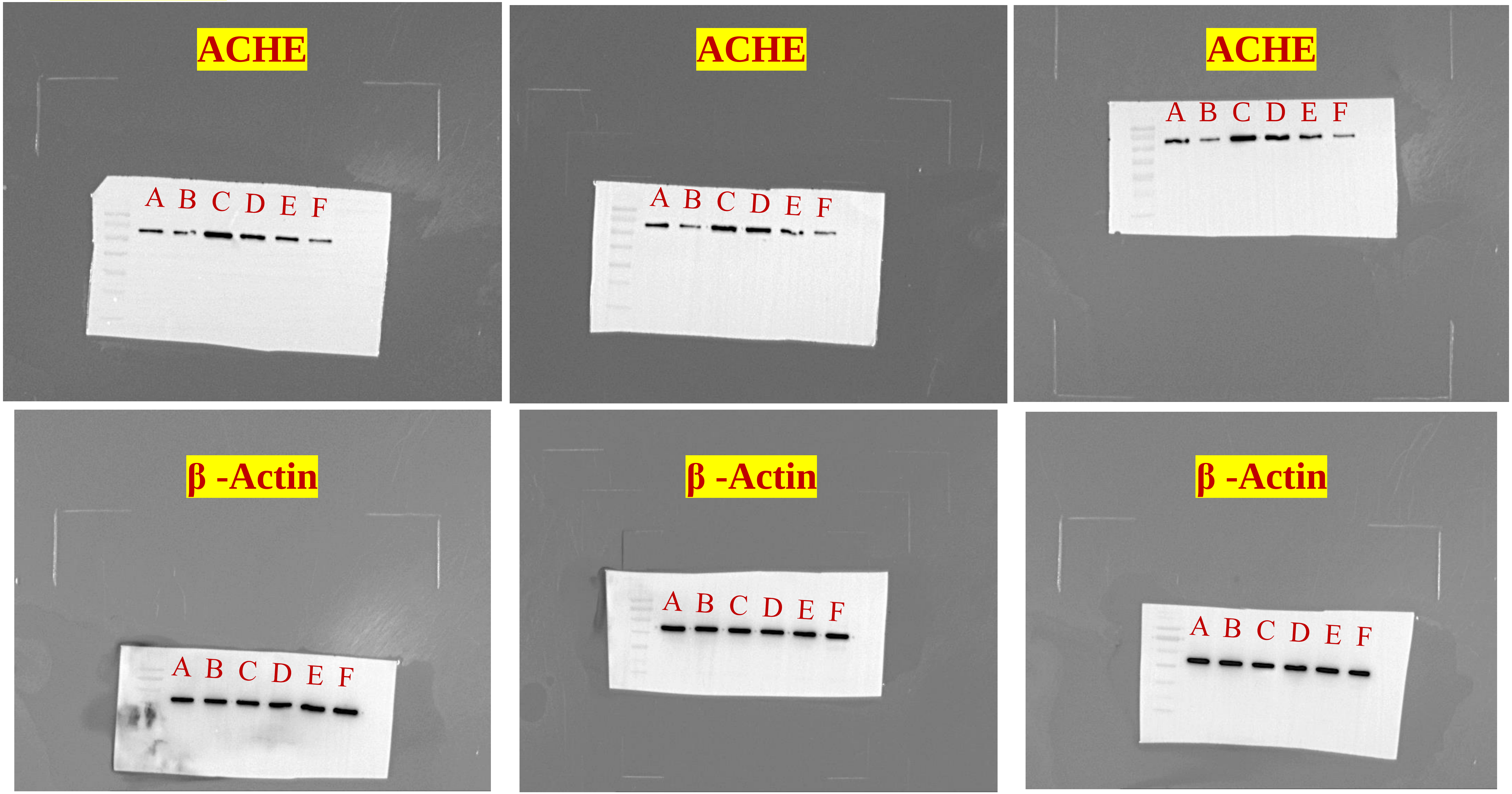
ACHE
A B C D E F
ACHE
A B C D E F
ACHE
A B C D E F
β -Actin
A B C D E F
β -Actin
A B C D E F
β -Actin
A B C D E F

# Figure 5F

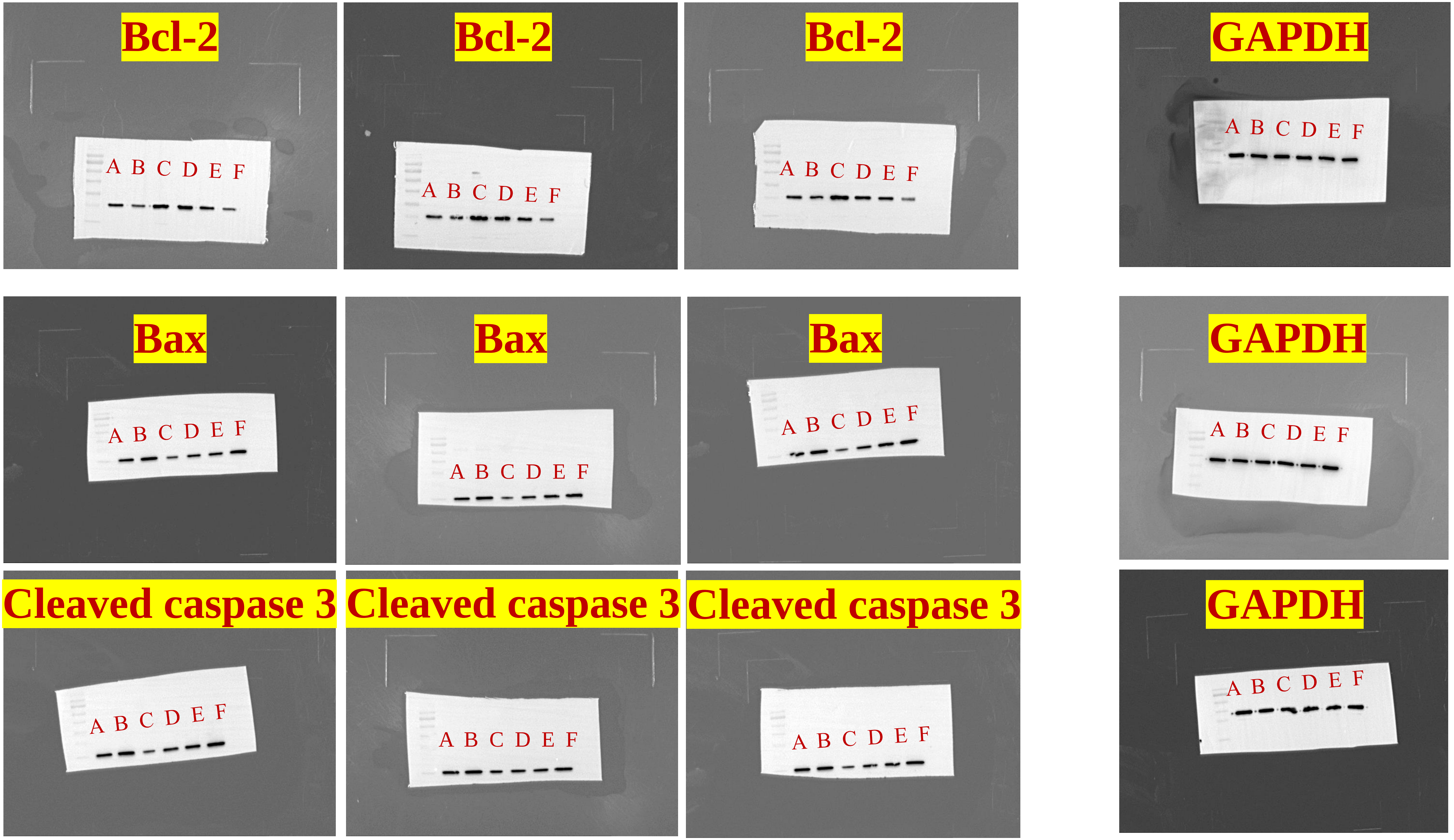
Bcl-2
A B C D E F
Bcl-2
A B C D E F
Bcl-2
A B C D E F
GAPDH
A B C D E F
Bax
A B C D E F
Bax
A B C D E F
Bax
A B C D E F
GAPDH
A B C D E F
Cleaved caspase 3
A B C D E F
Cleaved caspase 3
A B C D E F
Cleaved caspase 3
A B C D E F
GAPDH
A B C D E F

Figure 5G

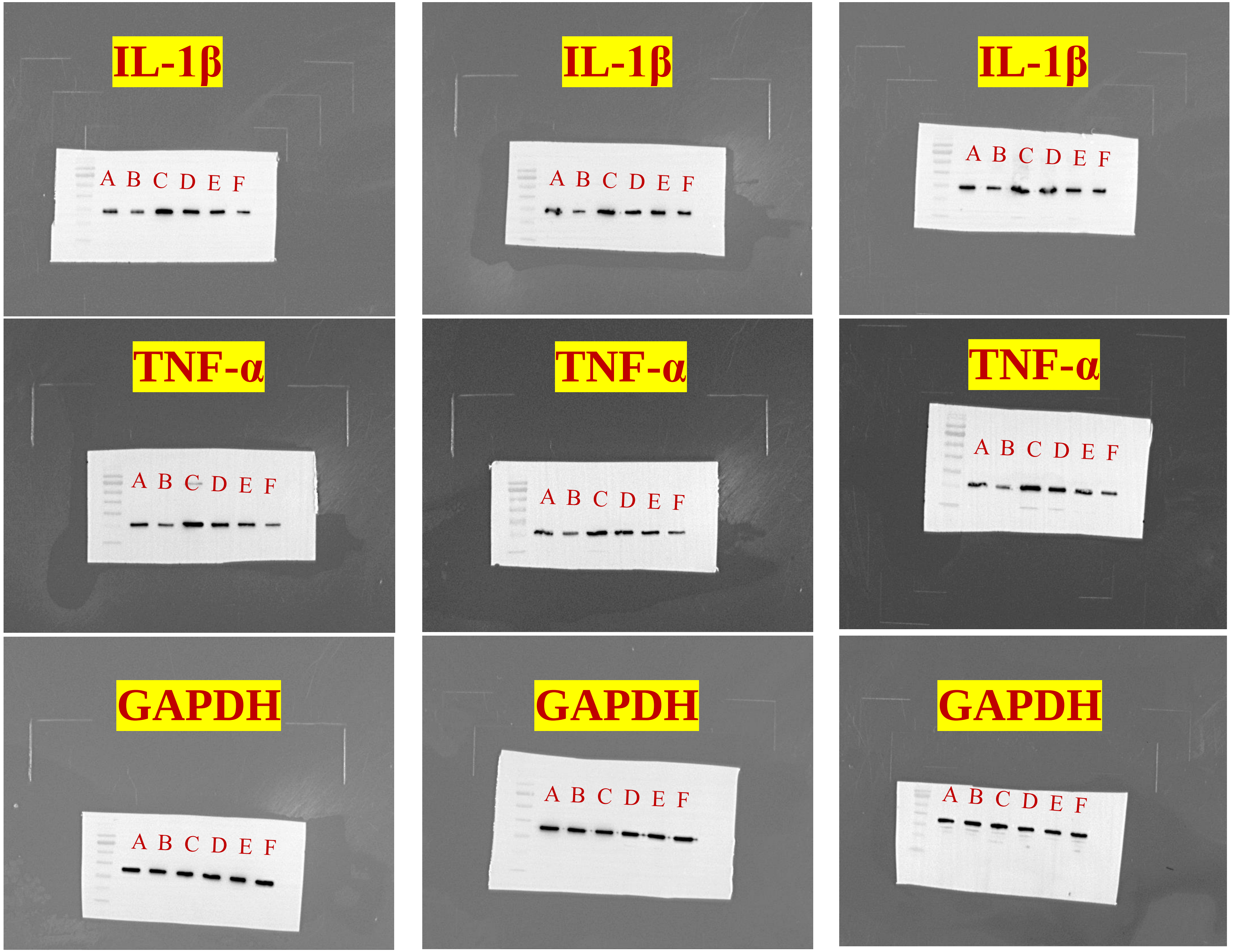
IL-1β
A B C D E F
IL-1β
A B C D E F
IL-1β
A B C D E F
TNF-α
A B C D E F
TNF-α
A B C D E F
TNF-α
A B C D E F
GAPDH
A B C D E F
GAPDH
A B C D E F
GAPDH
A B C D E F
